# Valproic Acid‐Induced Autistic‐Like Behavior Is Accompanied by Intestinal Damage Driving Changes in Gut Permeability in a Sex‐Dependent Way in Rats

**DOI:** 10.1111/jnc.70316

**Published:** 2025-12-15

**Authors:** Bruna Longo, Ruan Kaio Silva Nunes, Camila André Cazarin, Thiago Farias de Queiroz e Silva, Joanna Sievers, Ana Caroline dos Santos Nilz, Larissa Venzon, Levy Mota da Silva, Caio Henrique Willrich, Benhur Judah Cury, Regina Azevedo Costa, Márcia Maria de Souza, Cristina Aparecida Jark Stern, Aleksander Roberto Zampronio, Luisa Mota da Silva

**Affiliations:** ^1^ Department of Pharmacology Federal University of Santa Catarina Florianópolis Santa Catarina Brazil; ^2^ Postgraduate in Pharmaceutical Sciences University of Vale do Itajaí Itajaí Santa Catarina Brazil; ^3^ Department of Pharmacology Federal University of Paraná Curitiba Paraná Brazil

**Keywords:** autism spectrum disorder, intestinal permeability, sexual dimorphism, valproic acid

## Abstract

A significant proportion of individuals with autism spectrum disorder (ASD) experience gastrointestinal disturbances exacerbating behavioral symptoms. Therefore, this study investigated alterations in the intestinal mucosa that influence intestinal permeability in rats exposed to valproic acid (VPA) in utero, as well as the sexual differences in it. After assessing social behavior, intestinal permeability was assessed using FITC‐dextran, and vascular permeability was evaluated through Evans blue dye tests in the intestine and blood–brain barrier (BBB) of male and female offspring. Furthermore, microscopic analyses were performed to assess mucosal architecture and quantify mucin levels, while inflammatory and oxidative parameters, 5‐HT and 5‐HIAA levels, and serum lipopolysaccharide (LPS) concentrations were measured. Males, but not females, exposed to VPA exhibited reduced social interaction time. Only VPA males showed increased intestinal and vascular permeability, histological changes, elevated mucin levels, and higher serum LPS levels. Male, but not female, rats exposed to VPA displayed increased BBB permeability, and both showed reduced intestinal muscular layer thickness. Additionally, males showed increased levels of reactive oxygen species (ROS) and malondialdehyde (MDA) and enhanced glutathione *S*‐transferase (GST) and myeloperoxidase (MPO) activities. Conversely, females exhibited no elevation in ROS or MDA intestinal levels. Moreover, a reduction in 5‐HT turnover was evidenced in VPA‐females compared to VPA‐males. These findings support the validity of this model as a tool for investigating the role of intestinal barrier dysfunction in ASD and for identifying novel pharmacological targets in this field, considering the sexual differences in search of the practice of personalized and precision medicine.

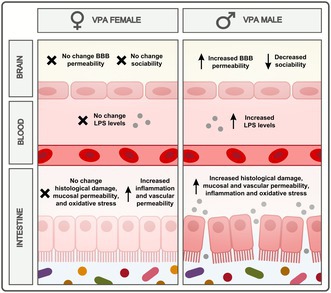

## Graphical abstract

Exposure to valproic acid (VPA) caused sex‐dependent changes in the gut–brain axis, with more pronounced effects in males, including intestinal barrier dysfunction, blood–brain barrier (BBB) impairment, and social behavior deficits. Females showed intestinal inflammation and increased vascular permeability in the ileum, but no behavioral or intestinal barrier impairments.

**Abbreviations**
5‐HIAA: 5‐hydroxyindoleacetic acid
5‐HT: 5‐hydroxytriptamine
AB: Alcian blue
ANOVA: analysis of variance
ASD: autism spectrum disorder
BBB: blood–brain barrier
CAS: chemical abstracts service
CNS: central nervous system
ELISA: enzyme‐linked immunosorbent assay
FITC: fluorescein isothiocyanate
GD: gestational day
GI: gastrointestinal
GSH: glutathione
GST: glutathione *S*‐transferase
HE: hematoxylin/eosin
HPLC: high‐performance liquid chromatography
LPS: lipopolysaccharide
MAPK: mitogen‐activated protein kinase
MDA: malondialdehyde
MPO: myeloperoxidase
PAS: periodic acid‐schiff
PBS: phosphate‐buffered saline
PND: postnatal day
ROS: reactive oxygen species
SI: social interaction
TLR4: toll‐like receptor 4
VPA: valproic acid

## 1 Introduction

Autism spectrum disorder (ASD) is a neurodevelopmental illness marked by difficulties communicating, socializing, and engaging in limiting or repetitive activities and interests (Lord et al. 2020). In addition to neurobehavioral symptoms, ASD is linked to a variety of comorbidities, including gastrointestinal disorders (Al‐Beltagi 2021). Individuals with ASD are predicted to be four times more likely to suffer from gastrointestinal (GI) issues than the neurotypical population (McElhanon et al. 2014). Constipation, diarrhea, abdominal pain, nausea, and gastroesophageal reflux are some of the most reported GI symptoms (Chaidez et al. 2014; Chandler et al. 2013; Kamionkowski et al. 2022).

In recent years, the role of GI in ASD has gained significance, particularly because it appears to increase neurological behaviors such as maladaptive behaviors, sleep issues, anxiety, irritability, self‐injury, and the severity of social and sensory symptoms (Pusponegoro et al. 2015; Restrepo et al. 2020; Kreider et al. 2021; Mazefsky et al. 2014). In this setting, the role of abnormal communication in the gut–brain axis in ASD has been explored, primarily through experimental models. Sgritta et al. (2019) describe a vagus nerve‐dependent mechanism in which *Lactobacillus reuteri* enhances social behavior in Shank 3−/− mice, a genetic ASD model. Despite this, Sgritta et al. (2019) did not report changes in intestinal permeability in Shank 3−/− mice. However, the leaky gut—a term that refers to increased intestinal permeability—has been hypothesized as an explanation for how GI changes affect the CNS in ASD (Dargenio et al. 2023).

Rats exposed to valproic acid (VPA) prenatally show abnormalities in brain structure and behavioral deficits comparable to those seen in ASD patients (Gąssowska‐Dobrowolska et al. 2020; Hirsch et al. 2018). As a result, intrauterine VPA exposure in rats has been widely employed as a model of ASD and thus a viable method for ASD research. Aside from the significance of VPA exposure's central nervous system (CNS) repercussions, the scientific community has recently highlighted the gastrointestinal abnormalities observed in this autism‐like model, supporting it as a good tool for investigating the gut comorbidities that arise in ASD.

In this context, Liu et al. (2018) described that the VPA‐induced autism‐like model in rats also mimics the microbiome features of autism, just as it has also been proven that therapeutic interventions able to modulate the intestinal microbiota can improve behavioral changes in male rats of the VPA autism‐like model (Vasconcelos et al. 2024). Besides, it was already described that VPA exposure during pregnancy led to pathological maternal intestinal changes, resulting in alterations in maternal gut microbiota, suggesting that GI problems in ASD may be associated with maternal intestinal inflammation and microbiota abnormality (Li et al. 2024). Moreover, the alterations in the gut microbiota and fecal metabolites in intrauterine VPA‐exposed rats showed sex‐specific differences (Gu et al. 2021), which are related to the autism‐like behavior (Gu et al. 2022) driving the need for ASD therapy to consider the differences between the sexes in the future.

Interestingly, therapies involving probiotics and/or prebiotics have demonstrated encouraging benefits in reducing the autistic‐like phenotype in male rats exposed to intrauterine VPA and reducing alterations in the brain, including 5‐hydroxytriptamine (5‐HT) modulation (Adıgüzel et al. 2022; Yang et al. 2024) suggesting that microbial manipulation with probiotics and prebiotics could be a promising treatment for autism‐like symptoms. Together with changes in microbiota, some studies have been describing the gastrointestinal damage in rats exposed prenatally to VPA. Varley and Browning (2024) performed functional studies that showed that both male and female VPA offspring had a delay in gastric emptying. In addition, using eosin and hematoxylin staining, Salari et al. (2024) showed that histopathological consequences in the small intestine were more significant in the VPA‐exposed male animals.

Despite the recent emphasis on gastrointestinal changes in the VPA‐induced autism‐like phenotype, many elements of GI dysfunction in ASD patients remain unclear, including the leaky gut. As a result, the current study postulated that the VPA‐induced autistic‐like model may be an appropriate model for studying the leaky gut function in ASD, evidencing that the presence of bacterial products from a highly permeable intestine in blood circulation contributes to autism‐like behavioral and biochemical alterations and that this model also allows for the verification of sex differences in this phenomenon.

## 2 Methods

### 2.1 Drugs and Reagents

The following substances were used: Valproic acid (CAS n° 1069‐66‐5, Sigma‐Aldrich), PBS (Product n° P3813, Sigma‐Aldrich), Evans blue (CAS n° 314‐13‐6, Sigma‐Aldrich), perchloric acid (CAS n° 7601‐90‐3, Sigma‐Aldrich), sodium metabisulfite (CAS n° 7681‐57‐4, Sigma‐Aldrich), 3,4‐dihydroxybenzylamine hydrobromide (CAS n° 16290‐26‐9, Sigma‐Aldrich), citric acid monohydrate (CAS n° 5949‐29‐1, Sigma‐Aldrich), octane‐1‐sulfonic acid (CAS n° 5324‐84‐5, Sigma‐Aldrich), ethylenediaminetetraacetic acid (CAS n° 60‐00‐4, Sigma‐Aldrich), methanol (CAS n° 67‐56‐1, Sigma‐Aldrich), 5‐HT (CAS n° 50‐67‐9, Supelco), 5‐hydroxyindoleacetic acid (CAS n° 54‐16‐0, Sigma‐Aldrich), potassium phosphate buffer (Product n° 1.99017, Supelco), trichloroacetic acid (CAS n° 76‐03‐9, Sigma‐Aldrich), Tris buffer (Product n° PPB023, Sigma‐Aldrich), 5,5′‐dithiobis (2‐nitrobenzoic acid) (CAS n° 69‐78‐3, Sigma‐Aldrich), sodium dodecylsulfate (CAS n° 151‐21‐3, Sigma‐Aldrich), FITC‐dextran (CAS n° 60842‐46‐8, Sigma‐Aldrich), acetic acid (CAS n° 64‐19‐7, Sigma‐Aldrich), thiobarbituric acid (CAS n° 504‐17‐6, Sigma‐Aldrich), hexadecyltrimethylammonium (CAS n° 57‐09‐0, Sigma‐Aldrich), sodium sulfate (CAS n° 127‐09‐3, Sigma‐Aldrich), bovine serum albumin (CAS n° 9048‐46‐8, Sigma‐Aldrich), 2′,7′‐dichlorodihydrofluorescein diacetate (CAS n° 4091‐99‐0, Sigma‐Aldrich), formaldehyde (CAS n° 50‐00‐0, Supelco), 1‐chloro‐2,4‐dinitrobenzene (CAS n° 97‐00‐7, Sigma‐Aldrich), and LPS kit (Cat n° EU3126, FineTest).

### 2.2 Animals

Wistar rats and their offspring were used. Initially, 12 females (120 days old, 200–220 g) and four males (120 days old, 250–300 g) from the Central Animal Laboratory of the University of Vale do Itajaí (UNIVALI) were used for mating. The animals were housed in propylene cages at 23°C ± 2°C under a 12‐h light/dark cycle with access to food and water *ad libitum*, housed with 4 to 5 animals per cage. The experiment protocols were approved by the UNIVALI Ethics Committee for the Use of Animals (approval number 002/22p1) and were carried out in accordance with the International Standards and the Ethical Guidelines on Animal Welfare.

At the end of the experiments, animals were euthanized by anesthetic overdose (thiopental 100 mg/kg) followed by exsanguination. After, clinical death was confirmed by the loss of consciousness, indicated by immobility and lack of response to non‐nociceptive and nociceptive stimuli. However, for procedures requiring blood collection, such as the intestinal permeability test and the assessment of bacterial lipopolysaccharide (LPS) levels and serotonin levels, the animals were anesthetized with ketamine (50 mg/kg, i.p.) and xylazine (10 mg/kg, i.p.).

### 2.3 Prenatal VPA‐Induced Autistic‐Like Behavior and Experimental Design

To obtain offspring with autistic‐like behavior, the methodology described by Schneider and Przewlocki (2005) was applied. Three females, with a controlled fertility cycle, were placed in the same box for each male during the mating period. Copulation was confirmed by the presence of a vaginal plug or sperm, observed using a vaginal swab. After confirmation of copulation, gestational day (GD) 0.5 was considered. Pregnant rats received a single 600 mg/kg intraperitoneal (i.p.) dose of valproic acid (VPA), administered on GD 12.5. On the other hand, females in the control group received saline solution (i.p.) at the same time. VPA was dissolved in saline solution at a concentration of 600 mg/mL.

Given the established literature on VPA's teratogenic effects (Schneider and Przewlocki 2005; Kim et al. 2011) the behavioral and neurodevelopmental outcomes in the offspring were evaluated as a priority, rather than evaluating teratogenicity per se. Nevertheless, some potential toxicity parameters were registered. Specifically, we monitored maternal weight gain, food intake, and mortality rate, as well as fetal weight, fetal viability, and birth outcomes, including reflex development and survival rate. Importantly, any animals that presented malformations that could affect locomotion or overall health were excluded from the study, ensuring that the results were not confounded by severe physical abnormalities.

The reproductive performance of VPA‐exposed and control (saline‐treated) rats was evaluated in this study, including the number of pups per litter, the number of males and females per litter, and pup body weight at postnatal day (PND) 7 (Figure S1, Appendix S1). To ensure that all pups received the same maternal care, the litter sizes were standardised by culling large litters to a maximum of 8 pups per litter, with an ideal sex ratio of 4 males and 4 females, as recommended by Schneider and Przewlocki (2005). However, in cases where litters were small or had an uneven sex ratio, priority was given to maintaining a consistent litter size across all experimental groups while ensuring that the sex ratio was as balanced as possible, like the approach used by Kim et al. (2011). Litter sizes and sex ratios were carefully documented, and the number of pups per litter was 11 in the control group (not exposed to VPA), with an average of 6 females and 5 males per litter; in pregnant rats treated with VPA, that is, those in the VPA group, the number of pups per litter was an average of 8, with 5 male pups born to 3 females. For the tests, 2 male pups and 2 female pups from each mother were used for each experiment to compose the experimental groups. The number of animals used in each set of experiments was 24, distributed as 12 (6 males and 6 females) in the control group and 12 (6 males and 6 females) in the VPA group. Three sets of independent experiments were made, totaling 72 rats in the study.

**FIGURE 1:**
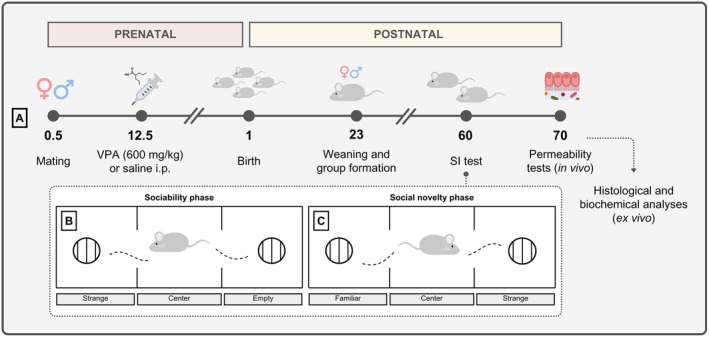
Experimental design. (A) Pregnant rats received a single dose of VPA (600 mg/kg, i.p.) or saline on gestational day 12.5. At PND 23, the offspring were weaned and separated into male and female groups (control or VPA). The sociability test (B) and social novelty preference test (C) were conducted on PND 60. On PND 70, animals underwent intestinal and blood–brain barrier (BBB) permeability assays, and samples were collected for histopathological, histochemical, and biochemical analyses.

On PND 23, the offspring from rats exposed to saline or VPA were weaned, separated by sex, and divided into control (*n* = 6 rats per group) and VPA groups (*n* = 6 rats per group). Behavioral tests to evaluate the social behavior of the offspring were conducted on PND 60. At the end of the behavioral tests, on PND 70, the animals were subjected to intestinal permeability and blood–brain barrier (BBB) tests. Samples of the ileum, colon, and blood were collected for histological and biochemical analyses. Females were tested regardless of estrous cycle stage. Figure 1 provides a representation of the experimental design used in this study.

### 2.4 Social Interaction (SI) Test

Sociability and preference for social novelties were assessed using the three‐chamber test and conducted as described in Elnahas et al. (2021). A rectangular acrylic box with three chambers (100 cm × 100 cm × 35 cm) was used, with equal sizes and partitions that allowed the animals free access between the chambers. Acrylic cylinders (diameter 15 cm; height 30 cm) containing several circular holes in their wall to allow physical interactions were placed in each of the side chambers to shelter strange animals. The test was carried out in three phases:

1. Habituation: Each animal was placed individually in the middle chamber and allowed to explore for 5 min. During the habituation phase, the acrylic cylinders in each of the side chambers were empty. This habituation phase helped to reduce anxiety and familiarize the animals with the testing environment, thereby minimizing any potential side biases. Corrections in room configuration and lighting are then made until the group of rats displays no side preference. Additionally, a counterbalanced design was employed, where the position of the social stimulus was randomized and alternated between the left and right chambers across trials. This approach ensured that any observed effects were not confounded by side preferences.
2. Sociability: Started after habituation, where a strange rat (same age and sex) was placed in the acrylic cylinders in one of the side chambers, while the acrylic cylinders in the opposite chamber were left empty. The subject rat was allowed to explore for 10 min. The time spent in each of the chambers and the time spent exploring (sniffing or touching) the strange rat were timed.
3. Social novelty preference: Started immediately after the sociability phase for a further 10 min. A new strange rat (same age and sex) was placed in the empty acrylic cylinders in the opposite chamber. This phase assesses the subject rat's preference for social novelty, so the time spent in each of the chambers and the time interacting with the familiar rat and the strange rat were timed.

Specifically, the time spent in each chamber, with a focus on the chamber containing the social stimulus (a conspecific animal) versus the chamber containing an inanimate object was recorded. The frequency and duration of social interactions, such as sniffing, approaching, and interacting with the social stimulus, also were measured. The experiment was conducted in a dark room with red light illumination, providing an intensity of 12 lx, in the morning between 7 and 10 a.m. The total test duration was 25 min: 5 min in the habituation phase, 10 min in the sociability test, and 10 min in the social novelty preference test. These behavioral parameters were quantified through video recordings made during the test sessions, and the images were then analyzed by trained observers.

After behavioral testing, the animals were euthanized with anesthetic injections (thiopental, 100 mg/kg, i.p.) for tissue collection for biochemical and histological analyses. Following the anesthetic overdose, exsanguination was performed as a secondary euthanasia method before tissue collection, and death was confirmed by the absence of consciousness.

### 2.5 Intestinal Permeability Test

The intestinal epithelial barrier was assessed using the fluorescein isothiocyanate (FITC)‐dextran test (4 KDa, Cat# FD4, Sigma‐Aldrich, St. Louis, MO, USA), as described by Jiang et al. (2021). Before the experiment, the animals were fasted for 8 h with access to water *ad libitum*. The animals were anesthetized with xylazine and ketamine (10 mg/kg and 50 mg/kg, i.p., respectively), and a longitudinal incision was made in the abdominal wall to allow access to the ileum. The ileum was exposed, and a 10 cm extension of the ileal tube was ligated at each end with silk thread. Afterwards, 1 mL of FITC‐dextran (10 mg/mL in PBS pH 7.4) was injected into the intestinal lumen, and the abdominal wall was sutured. After 30 min, blood was collected by cardiocentesis. The animals were euthanized by anesthetic deepening (thiopental, 100 mg/kg, i.p.) followed by exsanguination, and death was confirmed by the absence of consciousness. The blood collected was centrifuged (10 min, 12 000 g, 4°C), protected from light, and stored at −80°C for fluorimetric analysis. The fluorescence intensity of the serum was analyzed using a fluorescence spectrophotometer at an excitation wavelength of 485 nm and an emission wavelength of 528 nm. The values obtained were interpolated on a curve of FITC‐dextran diluted in PBS pH 7.4 at concentrations of 0 to 31.25 μg/mL.

### 2.6 Vascular Permeability Test

Vascular permeability in the ileum, colon, and BBB was measured by Evans blue dye extravasation, as described by Goldim, Dalla‐Giustina, and Petronilho (Goldim et al. 2019), with some modifications. The animals were anesthetized with xylazine and ketamine (10 mg/kg and 50 mg/kg, i.p., respectively), and Evans blue (1%, w/v, 3 mL/kg) prepared in saline solution was injected systemically (intravenously) through the tail vein. After 2 h of dye administration, the animals were euthanized using anesthetic deepening (thiopental, 100 mg/kg, i.p) followed by exsanguination After 30 min, blood was collected by cardiocentesis. The animals were euthanized by anesthetic deepening (thiopental, 100 mg/kg, i.p.) followed by exsanguination, and death was confirmed by the absence of consciousness. Samples of the ileum (10 cm) and the brain were removed. The structures were weighed, and the dye was extracted by incubation with formamide (2 mL/g tissue) for 24 h, according to the method described by Da Silva et al. (2013). The absorbance of the extravasated dye was measured in a spectrophotometer at 620 nm. The results were calculated using a coefficient of 7.810/M/cm and expressed as μmol of Evans blue/g of tissue.

### 2.7 Histopathological and Histochemical Analysis

The animals were euthanized by an overdose of thiopental (100 mg/kg, i.p.). Subsequently, exsanguination was performed as a secondary method, and death was confirmed by the absence of consciousness. After tissue collection, the samples of the ileum and colon were fixed in a solution composed of 85% ethanol, 10% formaldehyde, and 5% acetic acid (v/v) for 24 h. Subsequently, they were dehydrated with alcohol and xylene, embedded in paraffin wax, sectioned at 5 μm, and stained with hematoxylin/eosin (HE) or, for histochemical analysis of mucin content, with periodic acid‐Schiff (PAS) and Alcian blue (AB), respectively.

The lesion score was assessed according to the criteria described by Camuesco et al. (2012) and Utrilla et al. (2015), considering the presence of epithelial loss, crypt integrity, cell infiltration, and edema. The ileal and colonic tissues were evaluated according to the characteristics, and a score ranging from 0 (healthy tissue) to 3 (severe damage), depending on the item, was assigned to each one. In the end, the score was added up. Table 1 clarifies the histology score.

**TABLE 1:** Criteria for microscopic lesion assessment.

Characteristics	Score	Criteria
Mucosal epithelium	0	Intact epithelial surface
	1	Loss of < 5% of the epithelial surface
	2	Loss of 5%–10% of the epithelial surface
	3	Loss of > 10% of the epithelial surface
Crypt integrity	0	Intact crypts
	1	Loss of < 10% of the crypts
	2	Loss of 10%–20% of the crypts
	3	Loss of > 20% of the crypts
Cellular infiltration and edema	0	None
	1	Mild
	2	Moderate
	3	Severe

*Note:* Adapted from Camuesco et al. (2012) and Utrilla et al. (2015).

The height of the villi (from the tip of the villus to the villus–crypt junction) and the depth of the crypts (depth of the invagination between the villi) were measured using the ImageJ program, as described by Boeing et al. (2020). Five villi and crypts were measured, and the means were used to express the results.

The thickness in μm of the tunica muscularis was measured as described by Kim et al. (2013). The perpendicular distance from the inner muscle layer to the outer muscle layer was measured using the ImageJ program. For this measurement, 6 animals per group were used, and for each animal, 3 sections of the intestine sample were evaluated, spaced regularly apart, to capture any potential variability along the length of the tissue. Furthermore, for each section, five randomly selected fields were evaluated under the microscope, and the means of the thickness in μm from each animal were used to express the results. The images were previously calibrated.

Finally, for the histochemical analysis, the sections were photographed using an optical microscope (400× magnification), and the mucins were quantified using the ImageJ program.

### 2.8 Determination of Lipopolysaccharide Levels

To determine LPS levels, the animals were anesthetized with xylazine and ketamine (10 mg/kg and 50 mg/kg, i.p., respectively), and blood was collected by cardiocentesis. The animals were euthanized by anesthetic deepening (thiopental, 100 mg/kg, i.p.) followed by exsanguination, and death was confirmed by the absence of consciousness. The blood was then centrifuged (10 min, 12 000 g, 4°C), and the levels of LPS in serum were measured by enzyme‐linked immunosorbent assay (ELISA), using ELISA kits from FineTest (Wuhan, China), according to the manufacturer's instructions. Measurements were performed in triplicate, and the results were expressed as μg/mL.

### 2.9 Determination of Serotonin Levels

The levels of serotonin in the ileum were determined as described by Costa et al. (2024). The animals were euthanized by an overdose of thiopental (100 mg/kg, i.p.), followed by exsanguination as a secondary method, and death was confirmed by the absence of consciousness. After collection, samples from the ileum were homogenized with 0.1 M of perchloric acid containing 0.02% sodium metabisulfite and an internal standard (50 ng/mL 3,4‐dihydroxybenzylamine) and centrifuged at 10000 × g for 20 min at 4°C. Then, 20 μL of the supernatant obtained was injected into the High‐Performance Liquid Chromatography (HPLC). The mobile phase consisted of 20 g of citric acid monohydrate, 200 mg of octane‐1‐sulfonic acid sodium salt, 40 mg of ethylenediaminetetraacetic acid, and 900 mL of deionized water filtered through a 0.45 μm filter. Methanol was added for a final composition of 10% methanol (*v*/v). Concentrations of 5‐HT and 5‐hydroxyindoleacetic acid (5‐HIAA) were assessed by reversed‐phase HPLC with electrochemical detection.

The system consisted of a Synergi Fusion‐RPC‐18 reverse phase column (inner diameter 150 × 4.6 mm, 4 μm particle size), which was equipped with a 4 × 3.0 mm pre‐column (SecurityGuard Cartridges Fusion‐RP), an electrochemical detector (ESA Coulochem III electrochemical detector) equipped with a guard cell (ESA 5020) with the electrode set at 350 mV and a double electrode analytical cell (ESA 5011A), and an LC‐20AT pump (Shimadzu, Kyoto, Japan) that was equipped with a manual Rheodyne 7725 injector with a 20 μL loop. The column was kept inside a temperature‐controlled oven (25°C, Shimadzu, Kyoto, Japan). The cell had two chambers in series. Each chamber included a porous graphite colorimetric electrode, a double counter electrode, and a double reference electrode. The oxidizing potentials were set at 100 mV for the first electrode and 450 mV for the second electrode. Neurotransmitters were detected at the second electrode. The peak areas of the standards were used to quantify the sample peaks. The assays were performed on triplicate samples, and the results were given in ng/g of tissue.

### 2.10 Determination of Oxidative and Inflammatory Parameters

Before tissue collection, animals were euthanized by an overdose of thiopental (100 mg/kg, i.p.), followed by exsanguination to ensure complete euthanasia, and death was confirmed by the absence of consciousness. The ileum and colon were collected and homogenized with 200 mM potassium phosphate buffer (pH 6.5). The homogenate was then used to determine the levels of reduced glutathione (GSH) (Sedlak and Lindsay 1968) and malondialdehyde (MDA) (Percário et al. 1994). The homogenate was then centrifuged at 9000 × g for 20 min (4°C); the supernatant was used to measure reactive oxygen species (ROS) (Da Silva et al. 2013) levels, while the precipitate was used to measure glutathione *S*‐transferase (GST) (Habig et al. 1974) and myeloperoxidase (MPO) activity (Bradley et al. 1982). Protein concentrations were determined using the Bradford method (Bio‐Rad, Hercules, CA, USA), according to the manufacturer's instructions, using bovine serum albumin (2.5–15 mg/mL) as a standard. The measurements were taken in triplicate, and the findings were expressed in μg of GSH/g of tissue for GSH levels, mmol/g of tissue for MDA levels, fluorescence intensity for ROS amount, mmol GSH/mg of protein for GST activity, and mD.O/mg of protein for MPO activity.

### 2.11 Statistical Analysis

All collected data were initially tested using the Shapiro–Wilk normality test to determine normal distributions. No blinding was performed during the experiments. Group sizes were equal by design but were unequal in one case due to experimental loss (a male VPA‐exposed animal that developed a detrimental deformity to its health and survival). We replaced this exclusion to retain the study's balance and power. Parametric data were expressed as means ± standard deviation of the means, and statistical analysis was obtained using the Student's *t*‐test or two‐way ANOVA followed by the Sidak test, when applicable. Outliers were considered values corresponding to more than 2 standard deviations in relation to the means. Results with multiple potential outliers were analyzed with a nonparametric test. Nonparametric tests were expressed as median with interquartile range and analyzed using the Mann–Whitney test. The analyses were carried out using the GraphPad Prism program version 8.0 (GraphPad Software, San Diego, USA). Values of *p* < 0.05 were considered significant. Based on the sample size study, the effect size and statistical power of this study were calculated using GPower 3.1.9.7 software, considering a significance level of *α* = 0.05 and a statistical power of 0.8. Based on a medium effect size estimate (Cohen's *d* = 0.8), the calculation indicated that at least six animals per group were needed.

## 3 Results

### 3.1 Prenatal Exposure to VPA Impairs the Social Behavior of Male Rats but Not Female Rats

**FIGURE 2:**
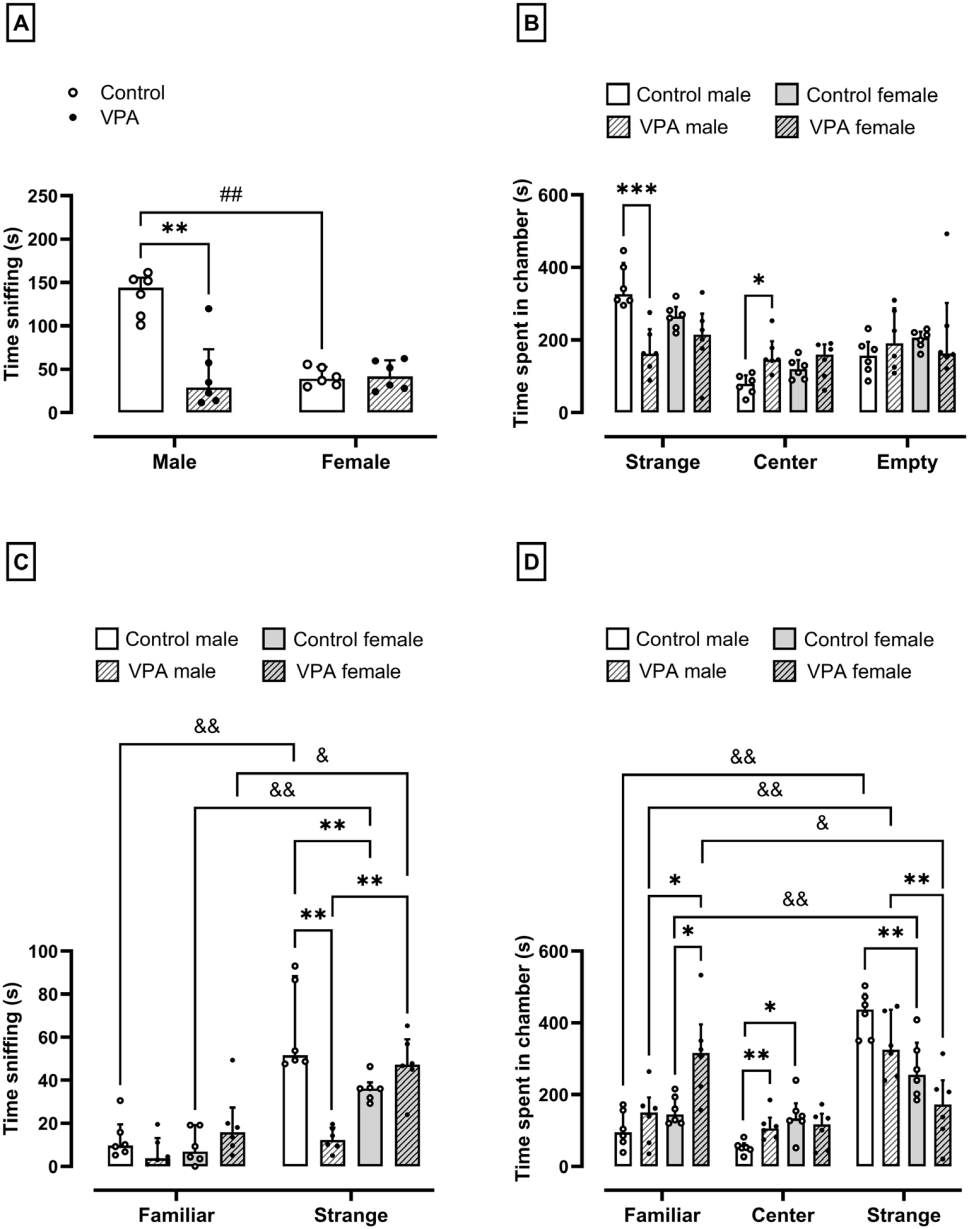
Effects of prenatal exposure to VPA on social interaction (A), time spent in chambers in the social interaction phase (B), preference for social novelty (C), and time spent in chambers in the preference for social novelty phase (D) in male and female rats. The results were expressed as medians with interquartile range (*n* = 6 rats per group), and the data were analyzed using the Mann–Whitney test. *p < 0.05, **p < 0.01, or ***p < 0.001 when compared to the control group. ^##^ *p* < 0.01 when compared to the female group. ^&^ *p* < 0.05 and ^&&^ *p* < 0.01 when compared to the strange group. VPA, valproic acid.

We evaluated the social behavior of rats exposed and not exposed to intrauterine VPA using the three‐chamber test. In the sociability test, male rats in the VPA‐exposed group showed a 68% reduction in the time spent interacting with the strange rat when compared to the males of the control group (144.18 ± 46.83 s, *p* = 0.008, U = 2.000, Figure 2A). As for the time spent in the chambers, there was a 46% reduction in the time spent in the strange rat's chamber (326.13 ± 150.12 s, *p* = 0.002, U = 0.000, Figure 2B) and a 52% increase in time spent in the center of the apparatus (79.20 ± 74.74 s, *p* = 0.004, U = 1.000, Figure 2B) compared to males in the control group. On the other hand, females in the VPA‐exposed group showed no reduction in social interaction time (Figure 2A) or time spent in the chambers (Figure 2B) in the sociability test when compared to the rats in the control group. Interestingly, in the sociability test, control females showed a significant reduction in time spent interacting with the strange rat compared to the control male rats (144.18 ± 46.83 s, *p* = 0.002, U = 0.000, Figure 2A).

In the social novelty preference test, males in the VPA‐exposed group showed no differences between the time spent interacting with the familiar and strange rat, contrary to the findings regarding the time spent interacting between the familiar and strange rat in the control group (*p* = 0.002, U = 0.000, Figure 2C). However, when the time spent interacting with the strange rat was compared between the VPA and control groups, the offspring of males exposed to VPA showed a 79% reduction in the time spent interacting with the strange rat compared to the control group (51.57 ± 45.51 s, *p* = 0.002, U = 0.000, Figure 2C). In addition, males in the VPA group showed no differences in time spent in the strange rat chamber compared to males in the control group (Figure 2D). Also, in the social novelty preference test, females in the VPA group showed a 60% increase in time spent interacting with the strange rat compared to the familiar rat (15.86 ± 44.38 s, *p* = 0.02, U = 4.000, Figure 2C), which was like the interaction time of the control group rats with the unknown animal. However, although the females in the VPA group showed a preference for social interaction with the strange rat, there was a 43% increase in the time spent in the familiar rat's chamber compared to the control group (144.86 ± 95.88 s, *p* = 0.05, U = 5.000, Figure 2D).

### 3.2 Prenatal Exposure to VPA Promotes Intestinal Histological Damage in Male but Not Female Rats

**FIGURE 3:**
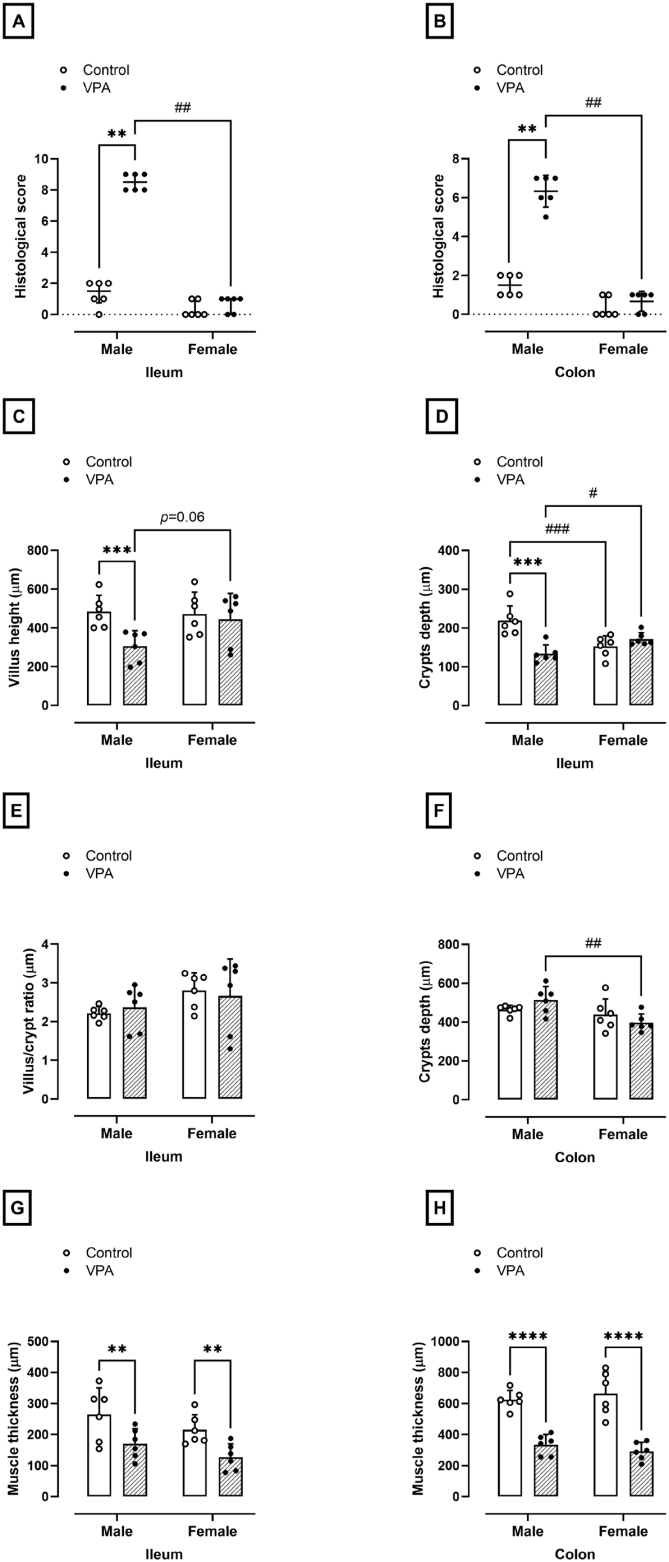
Effects of prenatal exposure to VPA on the histological score of the ileum (A) and colon (B), villus height (C), crypt depth (D), villus/crypt ratio (E) in the ileum, crypt depth (F) in the colon, muscle thickness in the ileum (G), and colon (H) of male and female rats. The parametric results (C–E) were expressed as means ± SD (*n* = 6 rats per group), and the data were analyzed using the two‐way ANOVA followed by the Sidak's multiple comparison test. The nonparametric results (A and B) were expressed as median with interquartile range (*n* = 6 rats per group), and the data were analyzed using the Mann–Whitney test. *p < 0.05, **p < 0.01, ***p < 0.001, or ****p < 0.0001 when compared to the control group. ^#^ *p* < 0.05, ^##^ *p* < 0.01, or ^###^ *p* < 0.001 when compared to the female group. VPA, valproic acid.

Males, but not females, in the VPA‐exposed group presented higher histological damage scores in the ileum (*p* = 0.002, U = 0.000, Figure 3A) and colon (*p* = 0.002, U = 0.000, Figure 3B), respectively, compared to the sex‐matched control group (1.5 ± 2 and 1.5 ± 1.0 scores, respectively). In the ileum, the increase in the histological damage score was accompanied by a 32% reduction in villus height (normality test *p* = 0.40, *F*_(1,10)_ = 15.93, Figure 3C) and a 40% reduction in crypt depth (normality test *p* = 0.32, Figure 3D, *F*_(1,10)_ = 23.21) in males in the VPA‐exposed group compared to the control group (483.78 ± 84.37 and 218.97 ± 37.88, respectively). However, no differences were found in the villus/crypt ratio (Figure 3E) and in the colon crypt depth (Figure 3F) between male rats exposed or not exposed to VPA. In females exposed to VPA, no changes were found in the villus height (Figure 3C), crypt depth (Figure 3D), and villus/crypt ratio (Figure 3E) in the ileum, as well as in the colon crypt depth (Figure 3F), compared to the female controls. However, the histological damage score in the ileum (1.5 ± 2 score, *p* = 0.002, U = 0.000, Figure 3A) and colon (1.5 ± 1 score, *p* = 0.002, U = 0.000, Figure 3B), and the ileum (normality test *p* = 0.32, *F*_(1,20)_ = 1.60, *p* = 0.04, Figure 3D) and colon (normality test *p* = 0.39, *F*_(1,10)_ = 5.92, *p* = 0.005, Figure 3F) crypt depth in the VPA‐female rats were significantly different compared to the VPA‐male rats.

In addition, a 35% reduction in the thickness of the tunica muscularis was observed in the ileum (normality test *p* = 0.97, 264.64 ± 85.44, *F*_(1,10)_ = 0.03, *p* = 0.001, Figure 3G) and 46% in the colon (normality test *p* = 0.98, 624.25 ± 60.84 μm, *F*_(1,10)_ = 4.14, *p* < 0.0001, Figure 3H) of males in the VPA‐exposed group compared to the control group. However, females in the VPA‐exposed group showed a reduction of 41% and 56% in the thickness of the tunica muscularis in the ileum (normality test *p* = 0.97, 215.60 ± 48.56, *F*_(1,10)_ = 0.03, *p* = 0.002, Figure 3G) and colon (normality test *p* = 0.98, 664.34 ± 60.84 μm, *F*_(1,10)_ = 4.14, *p* < 0.0001, Figure 3H), respectively, compared to the control group. Figure 5 shows representative images of these results.

### 3.3 Prenatal Exposure to VPA Promotes Increased Intestinal PAS‐ and AB‐Stained Mucins in Male but Not Female Rats

**FIGURE 4:**
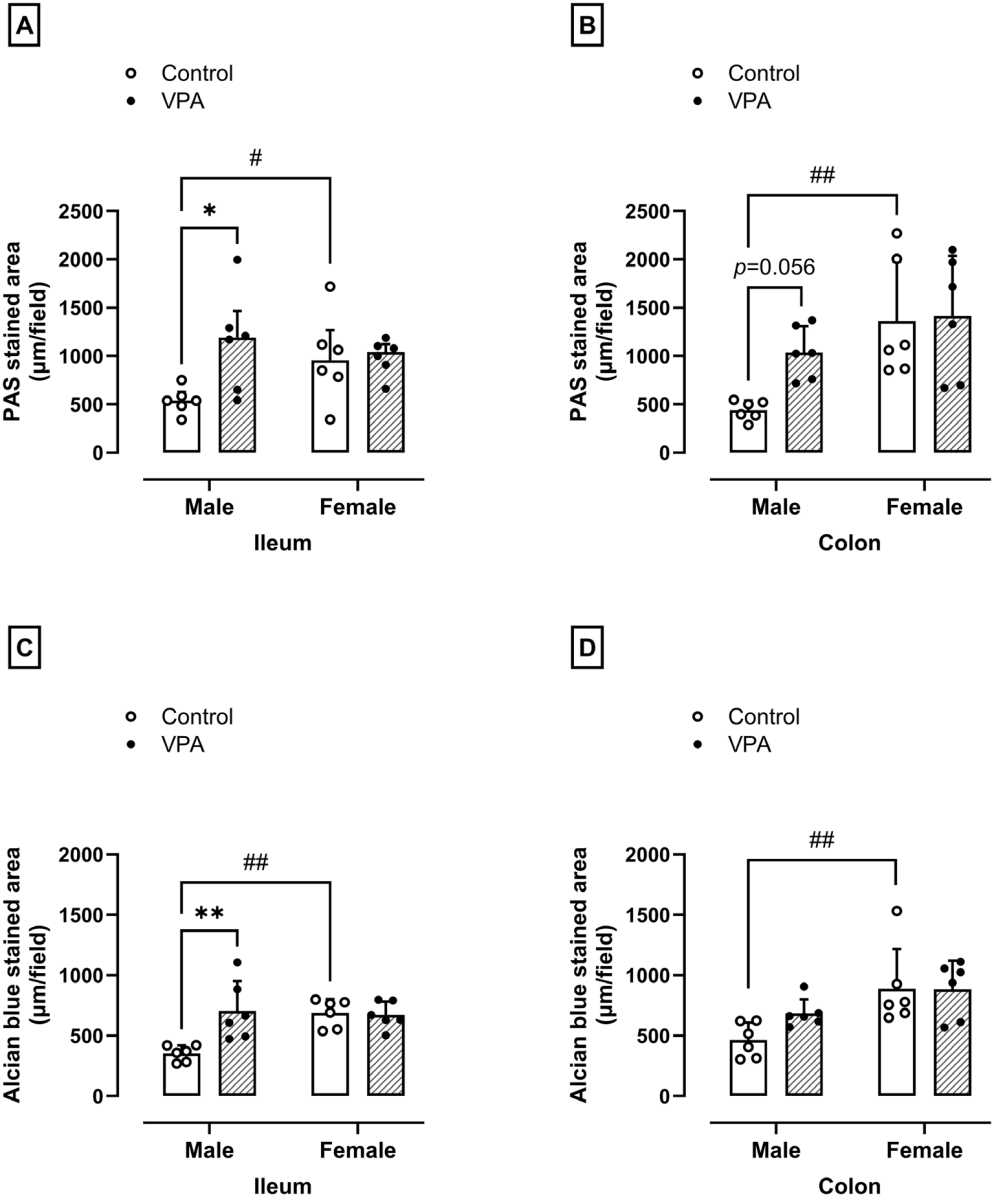
Effects of prenatal exposure to VPA on PAS‐stained mucins in the ileum (A) and colon (B), and Alcian Blue‐stained mucins in the ileum (C) and colon (D) of male and female rats. The parametric results (B–D) were expressed as means ± SD (*n* = 6 rats per group) and the data were analyzed using the two‐way ANOVA followed by the Sidak's multiple comparison test. The nonparametric results (A) were expressed as medians with interquartile range (*n* = 6 rats per group), and the data were analyzed using the Mann–Whitney test. *p < 0.05 or **p < 0.01 when compared to the control group. ^#^ *p* < 0.05 and ^##^ *p* < 0.01 when compared to the female group. VPA, valproic acid.

**FIGURE 5:**
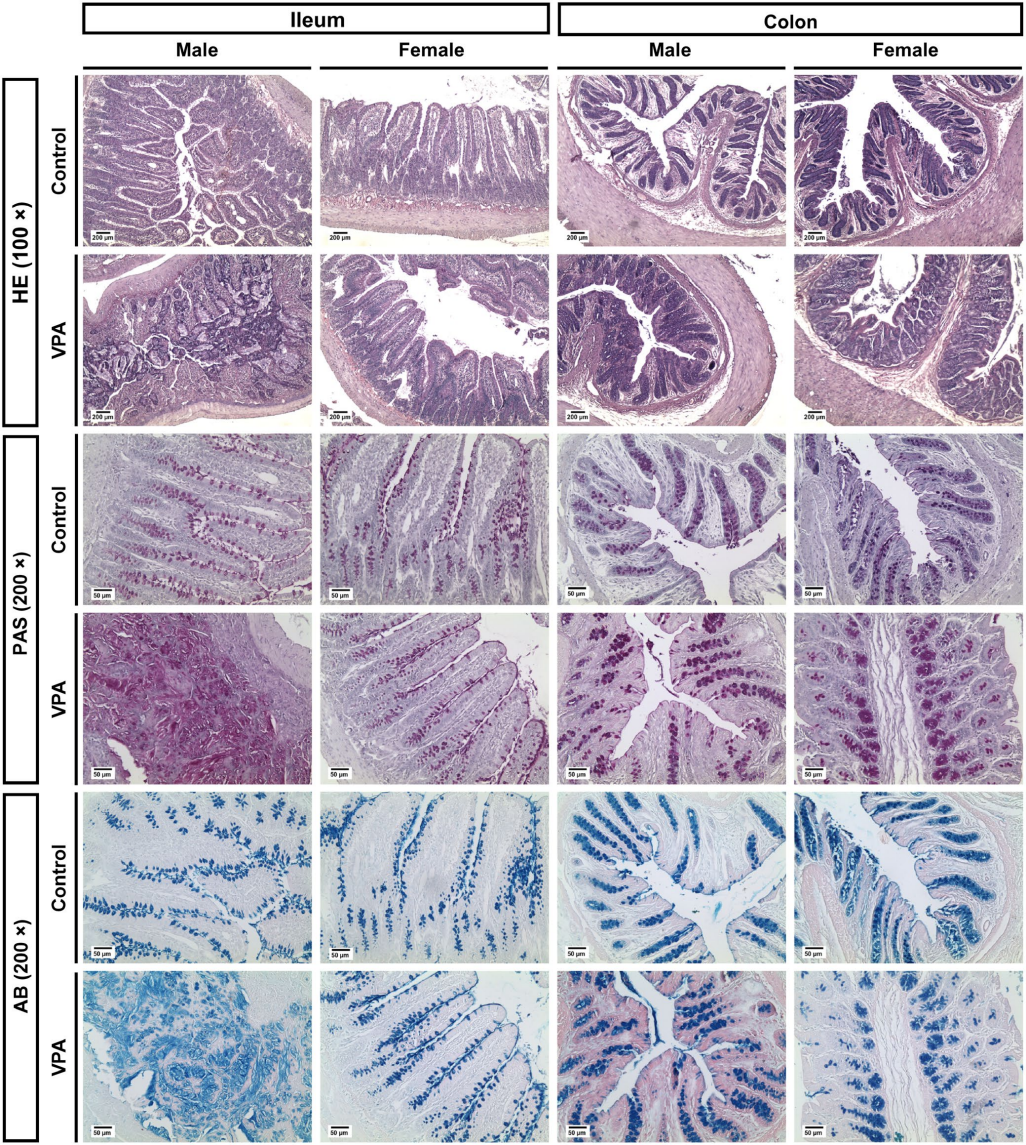
Representative images of the microscopic appearance of the ileum and colon of male and female rats from the control and VPA‐exposed groups. The tissues were stained with hematoxylin and eosin (HE), periodic acid Schiff (PAS), and Alcian Blue (AB). The images were recorded at 100× or 200× magnification. VPA, valproic acid.

In addition to histologic damage, males in the VPA‐exposed group showed a 2‐fold increase in PAS‐staining in the ileum (538.18 ± 178.97 μm/field, *p* = 0.02, U = 5.000, Figure 4A) and in the colon (normality test *p* = 0.82, *F*_(1,10)_ = 2.73, *p* = 0.05, Figure 4B), as well as in the AB‐staining in the ileum (normality test *p* = 0.36, *F*_(1,10)_ = 8.72, *p* = 0.005, Figure 4C) compared to males in the control group (539.68 ± 133.52, 440.14 ± 100.02, and 353.22 ± 65.75 μm/field, respectively). The values for AB‐stained mucins have not been changed in the colon of VPA‐exposed males (Figure 4D). In females, there were no differences in PAS‐stained mucins (Figure 4A,B) and AB‐stained mucins (Figure 4C,D) in the ileum and colon compared to females in the control group. However, the PAS‐stained mucins in the ileum (*p* = 0.04, U = 5.000, Figure 4A) and in the colon (normality test *p* = 0.82, *F*_(1,10)_ = 9.69, *p* = 0.004, Figure 4B) and the AB‐stained mucins in the ileum (normality test *p* = 0.36, *F*_(1,10)_ = 6.127, *p* = 0.002, Figure 4C) and colon (normality test *p* = 0.07, *F*_(1,10)_ = 11.78, *p* = 0.007, Figure 4D) of female control rats were significantly higher compared to the values found in the male control rats. Figure 5 shows representative images of PAS‐ and AB‐stained mucins of different experimental groups.

### 3.4 Prenatal Exposure to VPA Promotes Increased Intestinal Permeability in Male but Not Female Rats

**FIGURE 6:**
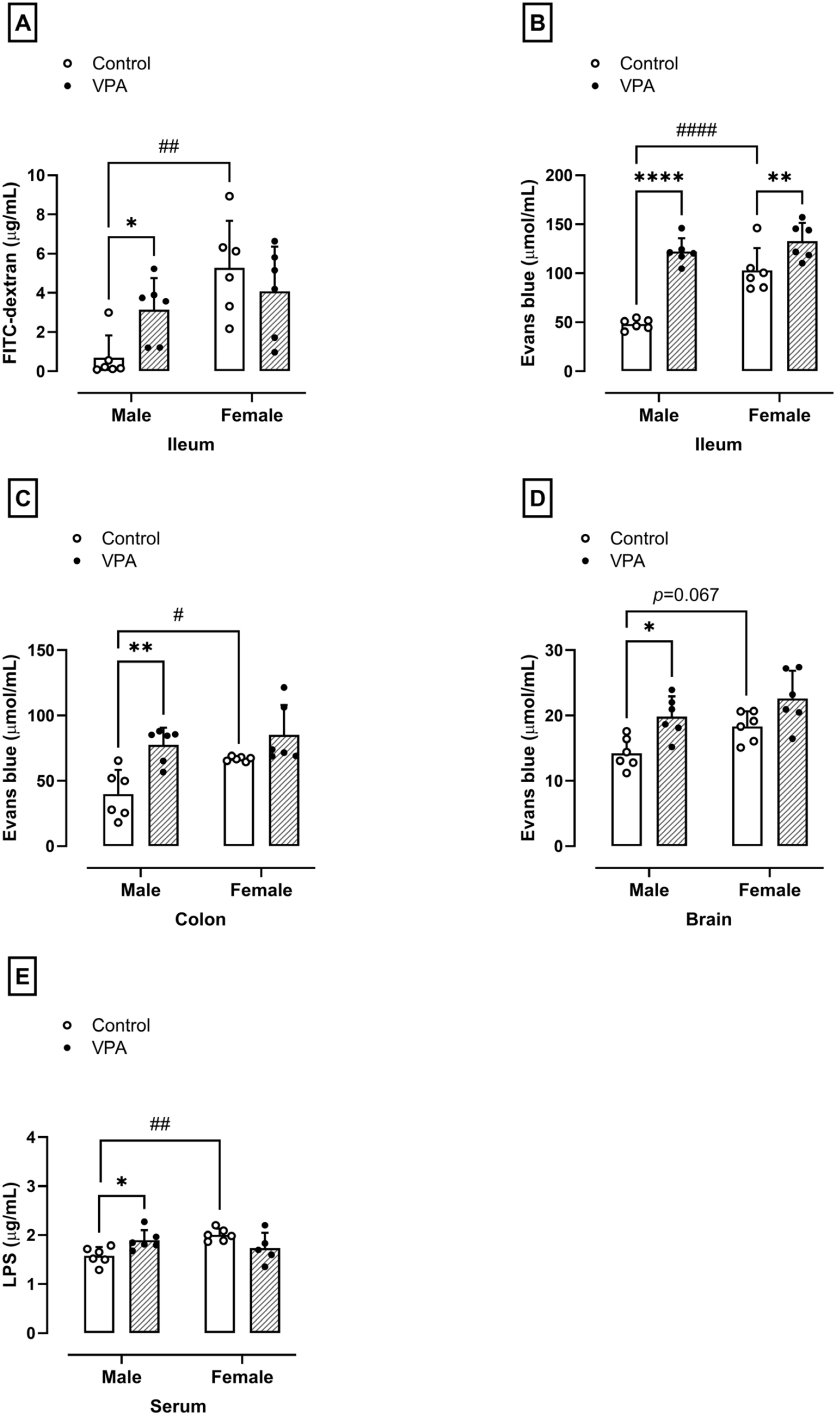
Effects of prenatal exposure to VPA on mucosal permeability in the ileum (A), vascular permeability in the ileum (B), colon (C), and blood–brain barrier (BBB, D), and serum lipopolysaccharide (LPS) levels (E) in male and female rats. The results were expressed as means ± SD (*n* = 6 rats per group), and the data were analyzed using the two‐way ANOVA followed by Sidak's multiple comparison test. *p < 0.05, **p < 0.01, or ****p < 0.0001 when compared to the control group. ^#^ *p* < 0.05, ^##^ *p* < 0.01, or ^####^ *p* < 0.0001 when compared to the female group. VPA, valproic acid.

The permeability of the ileum was evaluated by the capacity of FITC‐dextran to traverse the intestinal barrier and enter the bloodstream. It was observed that males in the VPA‐exposed group showed a 4.5‐fold increase in serum FITC‐dextran levels compared to males in the control group (normality test *p* = 0.77, 0.69 ± 1.14 μg/mL, *F*_(1,10)_ = 8.74, *p* = 0.03, Figure 6A). In contrast, females in the VPA‐exposed group showed no difference in serum FITC‐dextran levels compared to females in the control (Figure 6A). However, it was possible to note that the serum FITC‐dextran levels in the control female rats were significantly higher compared to control male rats (normality test *p* = 0.77, *F*_(1,20)_ = 12.33, *p* = 0.001, Figure 6A).

### 3.5 Prenatal Exposure to VPA Promotes Increased Vascular Permeability in the Intestine of Male and the BBB of Male and Female Rats

The vascular permeability was evaluated in the intestine and the BBB by the extravasation of Evans blue dye. In males of the VPA‐exposed group, vascular leakage of Evans blue dye increased 1.5 times in the ileum (48.17 ± 5.30 μmol/mL, normality test *p* = 0.09, *F*_(1,10)_ = 15.86, *p* < 0.0001, Figure 6B) and 68% in the colon (39.83 ± 18.60 μmol/mL, normality test *p* = 0.29, *F*_(1,10)_ = 2.04, *p* = 0.005, Figure 6C) compared to control rats, indicating an increase in vascular permeability. Females in the VPA‐exposed group showed a 29% increase in vascular permeability to this dye in the ileum (102.85 ± 22.77 μmol/mL, normality test *p* = 0.09, *F*_(1,10)_ = 15.86, *p* = 0.006, Figure 6B), but it did not change in the colon of these rats compared to the control group (66.86 ± 1.74 μmol/mL, normality test *p* = 0.29, Figure 6C). In addition, it was possible to note a significant increase in the vascular permeability in the ileum (normality test *p* = 0.09, *F*_(1,20)_ = 23.89, *p* < 0.0001, Figure 6B) and colon (normality test *p* = 0.29, *F*_(1,20)_ = 6.943, *p* = 0.01, Figure 6C) of the control female rats compared to control male rats. Moreover, males, but not females, in the VPA‐exposed group showed an increase in BBB permeability of 39% (normality test *p* = 0.81, *F*_(1,10)_ = 0.19, *p* = 0.04, Figure 6D) compared to the control group (14.24 ± 2.38 μmol/mL, respectively).

### 3.6 Prenatal Exposure to VPA Promotes Increased Serum LPS in Male but Not Female Rats

To assess whether intestinal barrier dysfunction in offspring exposed to prenatal VPA permitted the translocation of bacterial products, particularly LPS, into the systemic circulation. As shown in Figure 6E, the serum LPS levels were measured. It was observed that males (normality test *p* = 0.66, *F*_(1,19)_ = 2.30, *p* = 0.03), but not females, exposed to prenatal VPA showed a 19% increase in serum LPS levels compared to the control group (1.57 ± 0.18 and 2.02 ± 0.13 μg/mL, respectively). Furthermore, the LPS serum levels were increased in female control rats compared to the male control rats (normality test *p* = 0.66, *F*_(1,19)_ = 2.31, *p* = 0.005).

### 3.7 Prenatal Exposure to VPA Reduces 5‐HT Levels in the Ileum of Male Rats but Not in Females

**FIGURE 7:**
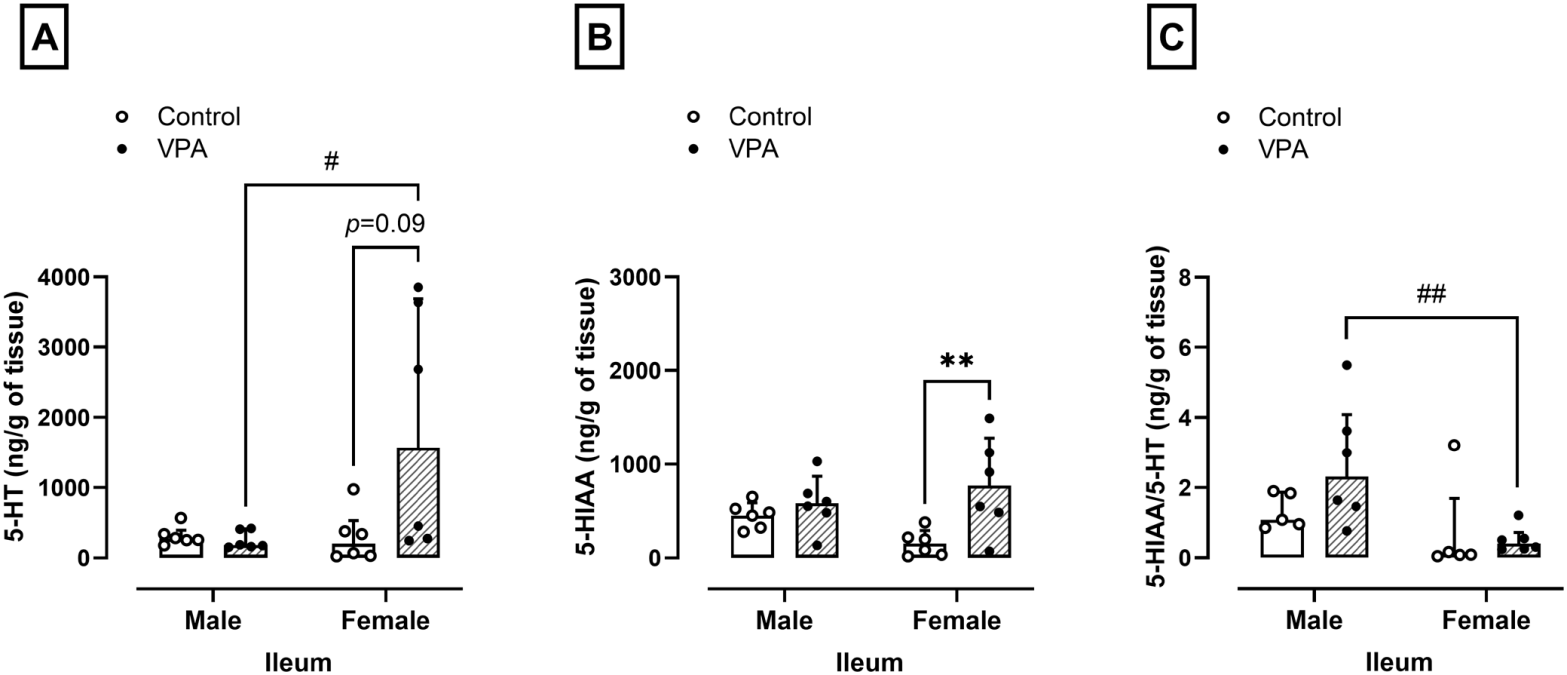
Effects of prenatal exposure to VPA on the levels of 5‐hydroxytryptamine (5‐HT, A), 5‐hydroxyindoleacetic acid (5‐HIAA, B), and 5‐HIAA/5‐HT turnover (C) in the ileum of male and female rats. The parametric results (B) were expressed as means ± SD (*n* = 6 rats per group), and the data were analyzed using the two‐way ANOVA followed by the Sidak's multiple comparison test. The nonparametric results (A and C) were expressed as medians with interquartile range (*n* = 6 rats per group), and the data were analyzed using the Mann–Whitney test. **p < 0.01 when compared to the control group. ^##^ *p* < 0.01 when compared to the female group. VPA, valproic acid.

Figure 7A–C show that there were no differences in the levels of 5‐HT, its metabolite 5‐HIAA, or the 5‐HT/5‐HIAA turnover in the ileum of male rats exposed to VPA compared to males in the control group (271.32 ± 160.7, 467.56 ± 241.65, and 1.08 ± 0.97 ng/g of tissue). In contrast, differences were observed in 5‐HIAA levels in the ileum of VPA‐exposed females as an increase of approximately 5 times greater than the female control group (141.50 ± 226.74 ng/g of tissue, normality test *p* = 0.58, *F*_(1,10)_ = 6.23, *p* = 0.002, Figure 7B) and VPA‐exposed females showed increased 5‐HT levels (1567.99 ± 3425.09 ng/g of tissue, *p* = 0.02, U = 4.000, Figure 7A) and reduced serotonin turnover compared to VPA‐exposed male rats (2.32 ± 2.80 ng/g of tissue, *p* = 0.004, U = 1.000, Figure 7C).

### 3.8 Prenatal Exposure to VPA Promotes Oxidative Stress in the Intestine of Male Rats and Intestinal Inflammation in Male and Female Rats

**FIGURE 8:**
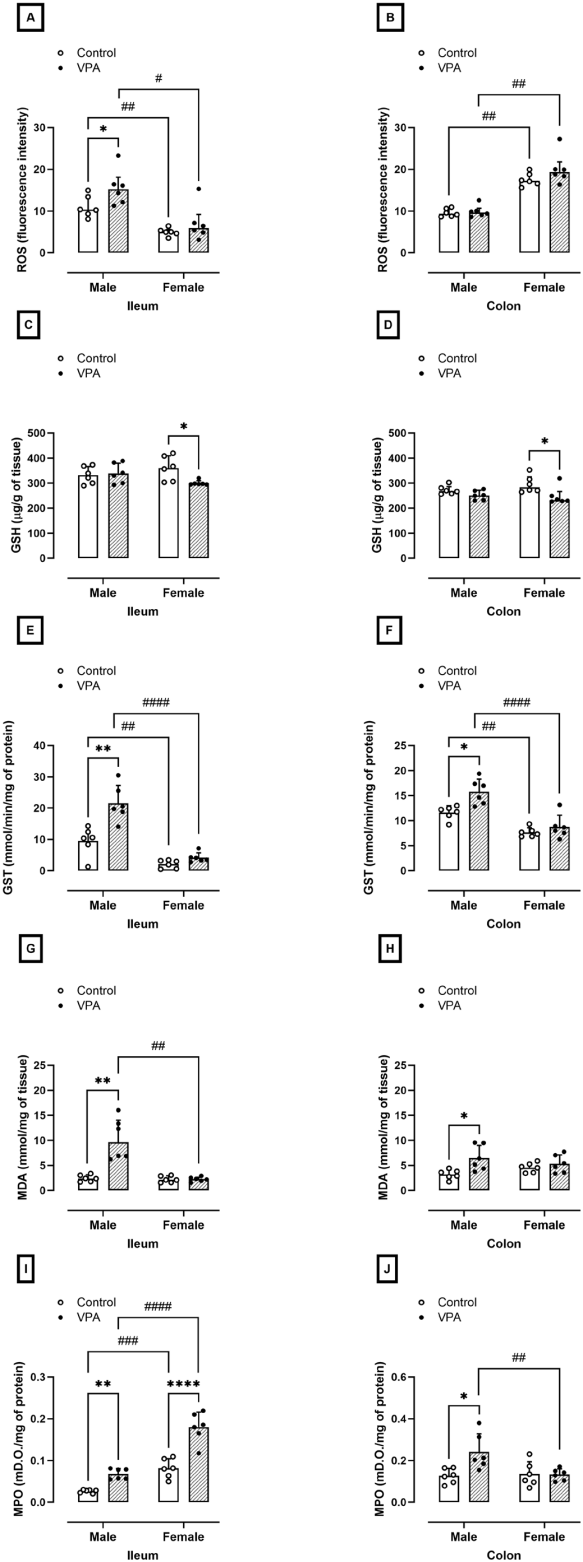
Effects of prenatal exposure to VPA on inflammatory and oxidative parameters in the ileum and colon of male and female rats. Reactive Oxygen Species (ROS, fluorescence intensity; A and B); Reduced glutathione (GSH, μg/mg of tissue; C and D); Glutathione S‐transferase (GST, mmol/min/mg of protein; E and F); Malondialdehyde (MDA, mmol/mg of tissue; G and H); Myeloperoxidase (MPO, mD.O/mg of protein; I and J). The parametric results (C, E, F, H, I, and J) were expressed as means ± SD (*n* = 6 rats per group) and the data were analyzed using the two‐way ANOVA followed by the Sidak's multiple comparison test. The nonparametric results (A, B, D, and G) were expressed as medians with interquartile range (*n* = 6 rats per group), and the data were analyzed using the Mann–Whitney test. *p < 0.05, **p < 0.01, or ****p < 0.0001 when compared to the control group. ^#^ *p* < 0.05, ^##^ *p* < 0.01, ^###^ *p* < 0.01 or ^####^ *p* < 0.0001 when compared to the female group. VPA, valproic acid.

Markers of oxidative stress and inflammation were evaluated in the ileum and colon of animals exposed to prenatal VPA. In the male VPA‐exposed group, we observed a 41% increase in ROS levels in the ileum (*p* = 0.04, U = 5.000) and no changes in the colon, compared to the control group (10.31 ± 4.78 and 9.34 ± 1.95 fluorescence intensity, Figure 8A,B, respectively). No differences were found in GSH levels in the ileum or colon of male VPA‐exposed rats compared to the control group (Figure 8C,D, respectively).

Regarding GST activity, GST activity increased by 125% in the ileum (normality test *p* = 0.08, *F*_(1,10)_ = 6.79, *p* = 0.002, Figure 8E) and 35% in the colon (normality test *p* = 0.38, *F*_(1,10)_ = 3.40, *p* = 0.01, Figure 8F), respectively, compared to the levels found in the control group (9.55 ± 4.53 and 11.62 ± 1.35 mmol of GSH/mg of protein, respectively). The MDA levels in the ileum (*p* = 0.002, U = 0.000, Figure 8E) and colon (normality test *p* = 0.69, *F*_(1,10)_ = 2.71, *p* = 0.02, Figure 7F) increased by 4 and 2 times in the male rats exposed to VPA, compared to the control group (2.37 ± 1.37 and 3.20 ± 0.94 mmol/g of tissue, respectively). In addition, MPO enzyme activity increased by 1.6 and 2 times in the ileum (normality test *p* = 0.15, *F*_(1,10)_ = 15.30, *p* = 0.005, Figure 8G) and colon (normality test *p* = 0.24, *F*_(1,10)_ = 5.18, *p* = 0.02, Figure 8H) of male rats exposed to VPA compared to males in the control group (0.026 ± 0.004 and 0.12 ± 0.03 mD.O./mg of protein, respectively).

On the other hand, females in the VPA‐exposed group showed an 18% and 16% reduction in GSH levels in the ileum (360.22 ± 49.06 μg/g of tissue, normality test *p* = 0.21, *F*_(1,10)_ = 5.27, *p* = 0.03, Figure 8C) and colon (283.79 ± 28.52 μg/g of tissue, *p* = 0.04, U = 5.000, Figure 8D) compared to females in the control group. However, no differences were detected in GST activity in the ileum (2.10 ± 1.31 mmol de GSH/mg of tissue, Figure 8E) and colon (7.65 ± 0.94 mmol de GSH/mg of tissue, Figure 8F) of female VPA‐exposed, compared to their control groups. In addition, we observed a 2.2‐fold increase in MPO activity in the ileum (0.08 ± 0.02 mD.O./mg of protein, normality test *p* = 0.15, *F*_(1,10)_ = 15.30, *p* = 0.0001) compared to females in the control group. No differences were found in ROS (Figure 8A,B) and MDA levels (Figure 8E,F) in the ileum and colon and in MPO activity in the colon (Figure 8H) of females in the VPA‐exposed group.

## 4 Discussion

The findings of the current study support the idea that rats with VPA‐induced autism are a good model to study intestinal alterations in the pathophysiology of ASD and that sexual dysmorphism may be an issue for this phenomenon. It is important to discuss the increased permeability of the BBB and digestive tract in ASD since it may lead to the development of novel treatment options considering sexual differences in the search for the practice of personalized and precision medicine.

**FIGURE 9:**
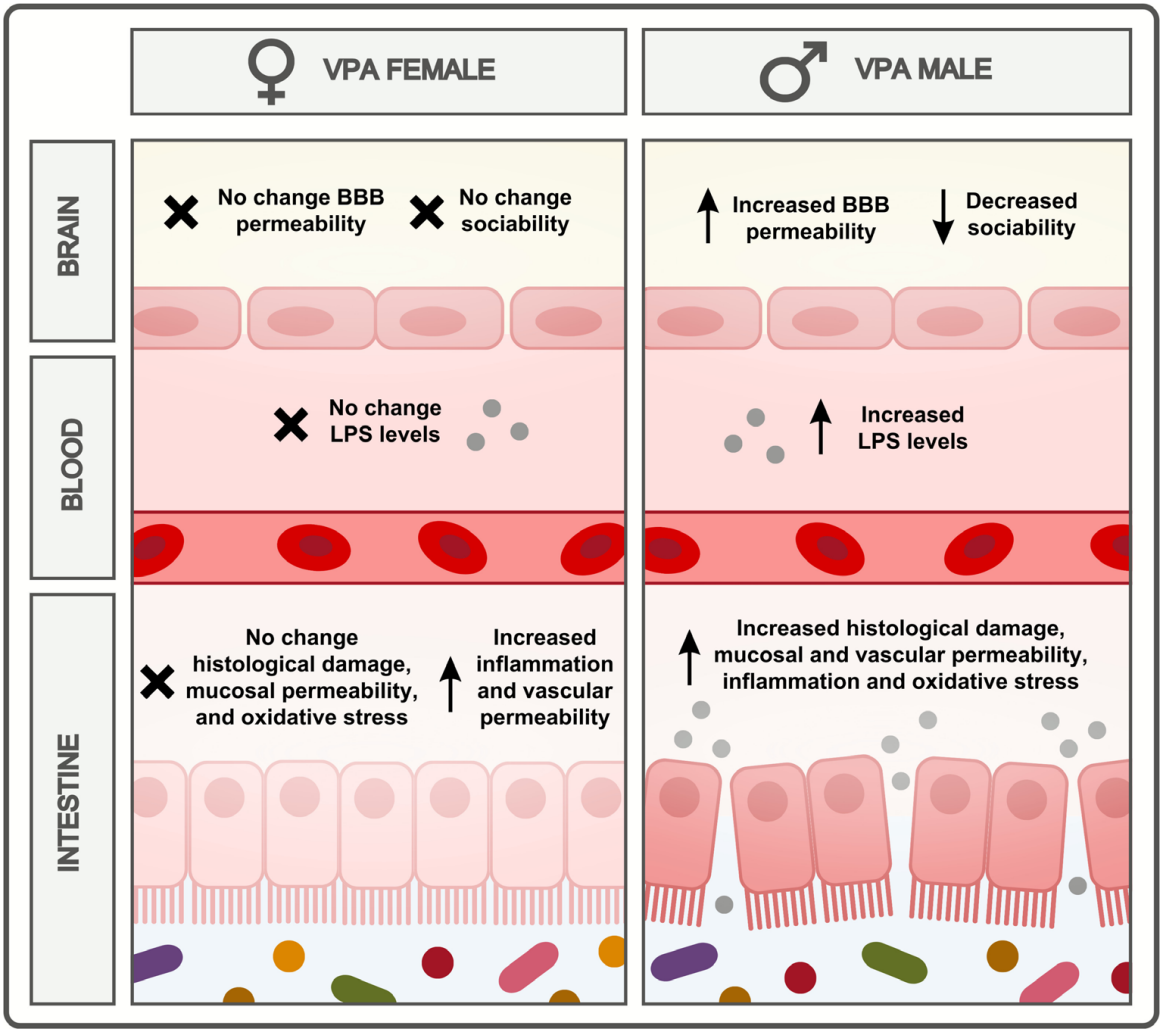
Graphical representation of sex‐related differences in intestinal changes in rats exposed to VPA in utero.

Indeed, our study demonstrates that prenatal exposure to VPA alters intestinal permeability and social behavior in a sex‐dependent manner, with males exhibiting more pronounced effects. The results show that VPA‐exposed male rats have impaired social behavior, as assessed by the three‐chamber test, and increased intestinal permeability, which is associated with alterations in the epithelial and muscular intestinal layers. These changes are accompanied by elevated levels of oxidative and inflammatory markers in the ileum and colon of VPA‐exposed males. The increased intestinal permeability is also associated with an increase in LPS in the circulation of male rats subjected to VPA, which may exacerbate the inflammatory response and contribute to the development of ASD symptoms. Additionally, it is possible that alterations in serotonin signaling in the intestine of VPA‐exposed rats may contribute to the dimorphism in intestinal changes in these animals (Figure 9).

The behavioral findings confirm the importance of considering sex differences in animal models of ASD. Previous studies have shown that ASD models, including the VPA model, exhibit social deficits in males (Chaliha et al. 2020), but not in females (Thabault et al. 2022; Nicolini and Fahnestock 2018; Tartaglione et al. 2019; Jaber 2023), when assessed by the three‐chamber test. These results are consistent with the observed impaired social behavior in VPA‐exposed males, while females do not exhibit significant deficits. However, recent studies suggest that females may exhibit complex social deficits that are not detected by traditional tests. For example, a study using a Live Mouse Tracker device that combines artificial intelligence and machine learning identified complex social deficits in VPA‐exposed females (Maisterrana et al. 2024). This indicates that females may exhibit different social deficits than males and that further investigations are needed to detect them.

The sex differences observed in ASD models are not limited to social behavior but also include changes in the cellular composition of some tissues. Females exhibit less loss of cerebellar Purkinje cells than males in ASD models (Tartaglione et al. 2019), suggesting that females may be better protected against developmental insults (Becker 2012; Haida et al. 2019). Together with the studies about the role of changes in CNS in autism, the study of the gut‐brain axis has shed light on the pathophysiology of ASD, and the VPA‐induced autism model has proven to be a valuable tool in this pursuit (Varley and Browning 2024; Zhong et al. 2023; Liu et al. 2024). In this context, this study assessed that cellular damage in intestinal tissue in rodents with autistic‐like behavior is also related to the biological sex.

A key finding of this research is that VPA‐exposed males exhibit significant intestinal damage, including intense epithelial damage and disturbances in crypt integrity, which compromise the intestinal epithelial barrier and lead to increased permeability. This “leaky gut” state may contribute to the development and exacerbation of ASD‐related signs and symptoms because it facilitates the presence of bacterial products in the blood circulation and, consequently, in the brain, mainly when the BBB is in a damaged state, culminating in neuroinflammatory insults (König et al. 2016; Longo et al. 2025).

The intestinal damage observed in VPA‐exposed males is consistent with previous studies, which have reported histological disturbances in the intestines of male animals (Varley and Browning 2024). However, the partial preservation of the intestinal epithelium in VPA‐exposed females suggests a sex‐dependent difference in the response to VPA exposure. Notably, while the intestinal epithelium is preserved in females, they do exhibit a reduction in the thickness of the muscular layer in the ileum, indicating some degree of intestinal damage. This damage may have implications for contractile and relaxing responses to the tissue and highlights the complexity of the gut‐brain axis in ASD.

The intestinal abnormalities in VPA‐exposed males are further characterized by an increase in mucus production evidenced by elevated levels of PAS‐ and AB‐mucin staining in the colon and ileum. In fact, the increased mucus production may be influenced by bacterial products, such as LPS and peptidoglycans (Gustafsson and Johansson 2022; Puricelli et al. 2022) and this expansion of the mucus layer may be an adaptive response to the detrimental effects of VPA on the intestinal tissue, given the defensive role of mucus (Li et al. 2022). Alternatively, it may be related to dysbiosis, a microbial composition imbalance that has been reported in ASD patients and VPA models (Li et al. 2022; Petersson et al. 2011). Notably, goblet cell function and mucus production are influenced by pathways that also mediate neuronal development in the brain (Herath et al. 2020), suggesting a potential link between intestinal and neurological abnormalities in ASD. These findings are consistent with previous studies showing alterations in goblet cell secretion in ASD‐like models (James et al. 2019; Franco et al. 2021) and highlight the need for further research on the role of goblet cells in ASD pathogenesis.

Consistent with clinical observations (Longo et al. 2025), the current study's results show that VPA‐exposed males exhibit increased intestinal permeability, as measured by the paracellular movement of FITC‐dextran from the intestinal lumen into the bloodstream. This increase in permeability is likely related to the damage observed in the intestinal mucosal barrier, including changes to the crypt‐villi and crypt structures in the ileum and colon. Furthermore, the study found that VPA‐exposed males also exhibit enhanced microvascular permeability in the ileum and colon, suggesting that dysfunction in endothelial cells lining the intestinal microvasculature occurs in conjunction with damage to the mucosal layers. This may facilitate the translocation of bacterial products into the systemic circulation, potentially contributing to the development of ASD symptoms. Further, these changes were observed only in males, highlighting the sex‐dependent differences in the response to VPA exposure.

The gut wall injury resulting from VPA exposure leads to the release of bacterial LPS into the bloodstream, which was evident in the increased levels of LPS observed in male VPA‐exposed rats. This increase in LPS may contribute to the development of intestinal permeability (Ghosh et al. 2014) and neuroinflammatory processes. The interaction between LPS and toll‐like receptor 4 (TLR4) may play a key role in this process, activating downstream signaling pathways that lead to the activation of mitogen‐activated protein kinase (MAPK) signaling pathways in microglial cells (Shimazu et al. 1999). The sex‐dependent difference in LPS levels achieved here, with males exhibiting increased levels and females not, may be related to the differences in intestinal permeability and damage observed between the sexes.

The VPA prenatal exposition promotes evident BBB permeability in brain regions related to the neocortex in male rats (Deckmann et al. 2021); however, this alteration remains unknown in female cohorts from VPA‐induced autism in rats. Previously, Fiorentino et al. (2016) described BBB dysfunctions in samples of the frontal cortex and cerebellum of male humans. The results achieved here also agree with these previous experimental and clinical data reporting a compromised BBB associated with ASD pathology, mainly in males.

Serotonin (5‐hydroxytryptamine, or 5‐HT) has been linked to ASD since research in the 1960s (Lee et al. 2012) and the potential role of 5‐HT in controlling intestinal membrane permeability is also an important consideration (Horie et al. 2022). In male rats, prenatal VPA treatment resulted in decreased 5‐HT levels and its higher metabolism in the CNS, and increased serum 5‐HT (Horie et al. 2022; Zhang et al. 2023). In addition, de Theije et al. (2014) found a decrease in 5‐HT levels in the ileum of male rats treated intrauterine with VPA.

In our results, two‐way ANOVA could not confirm the data of de Theije et al. (2014) about 5‐HT ileal levels in male VPA rats. However, despite the nonparametric distribution of the data acquired in ileal 5‐HT values, a *p* value of 0.09 was detected between the groups of females exposed and not exposed to VPA, accompanied by a significant increase in ileal HIIA levels in females exposed to VPA. Both findings need to be explored in future studies and may indicate that in females there is an increase in intestinal serotonergic signaling, which could consequently impact the greater resilience of these animals to the damage caused by VPA in the intestine, given that 5‐HT regulates intestinal epithelial homeostasis. Indeed, crypt epithelial cells express 5‐HT2A receptors (Fiorica‐Howells et al. 2002), 5‐HT antagonists can inhibit crypt cell proliferation (Tutton and Barkla 1980), and 5‐HT from enteric neurons promotes growth and turnover of the intestinal mucosal epithelium (Gross et al. 2012).

The disruption of the intestinal barrier and translocation of bacterial products, such as LPS, to the lamina propria can trigger or intensify the inflammatory process in the intestinal mucosa (Candelli et al. 2021; Wu et al. 2019). In this study, the increased MPO enzyme activity in the ileum and colon of males prenatally exposed to VPA corroborates the increased leukocyte infiltration observed in the histological findings of these animals, suggesting a significant inflammatory response. Notably, the increase in MPO activity was also observed in the ileum of females, although they did not show an increase in intestinal permeability, indicating that intestinal inflammation may be a complex process involving multiple factors.

The current literature suggests an imbalance of oxidative and antioxidative systems in autism (Bjørklund et al. 2020). Regarding this issue, the sex‐dependent effect on oxidative damage in the intestine of VPA animals is evident, with males showing increased levels of MDA and ROS in the ileum, while females do not exhibit any alterations. The decreased GSH availability in VPA male rats, particularly in the colon, may have contributed to the increased oxidative damage observed in these animals. In contrast, the preservation of GSH reserves in females may have played a crucial role in reducing oxidative damage in their intestines. The increased GST activity observed in both males and females exposed to VPA may be a compensatory mechanism to mitigate oxidative stress. Therefore, the findings of this study highlight the complex interplay between inflammation, oxidative stress, and antioxidant defenses in the intestine of VPA‐exposed animals.

## 5 Conclusion

The sex‐dependent differences in intestinal damage and permeability observed in this study have important implications for understanding the pathophysiology of ASD. The finding that males are more susceptible to intestinal damage and increased permeability may contribute to the development of more severe ASD symptoms, while the preservation of the intestinal epithelium in females may provide some protection against these effects. Further research is needed to fully elucidate the mechanisms underlying these sex‐dependent differences and to explore their potential therapeutic implications. To our knowledge, this is the first nonclinical study to demonstrate the influence of biological sex on intestinal permeability alterations in a VPA‐induced autism model. These findings support the validity of this model as a tool for investigating the role of intestinal barrier dysfunction in ASD and for identifying novel pharmacological targets in this field, considering the sexual differences in search of the practice of personalized and precision medicine.

## Author Contributions

**Bruna Longo:** investigation, formal analysis, data curation, writing – review and editing, writing – original draft. **Ruan Kaio Silva Nunes:** investigation, data curation, formal analysis. **Camila André Cazarin:** data curation, formal analysis, investigation. **Thiago Farias de Queiroz e Silva:** data curation, investigation. **Joanna Sievers:** data curation, investigation. **Ana Caroline dos Santos Nilz:** data curation, investigation. **Larissa Venzon:** investigation, data curation. **Levy Mota da Silva:** investigation, data curation. **Caio Henrique Willrich:** investigation, data curation. **Benhur Judah Cury:** investigation, data curation. **Regina Azevedo Costa:** investigation, data curation. **Márcia Maria de Souza:** supervision. **Cristina Aparecida Jark Stern:** supervision. **Aleksander Roberto Zampronio:** supervision. **Luisa Mota da Silva:** conceptualization, formal analysis, funding acquisition, resources, supervision, writing – original draft, writing – review and editing.

## Conflicts of Interest

The authors declare no conflicts of interest.

## Supporting information


**Appendix S1:** jnc70316‐sup‐0001‐AppendixS1.zip.

## Data Availability

The data that support the findings of this study are available from the corresponding author upon reasonable request.

## References

[jnc70316-bib-0001] Adıgüzel, E. , B. Çiçek , G. Ünal , M. F. Aydın , and D. Barlak‐Keti . 2022. “Probiotics and Prebiotics Alleviate Behavioral Deficits, Inflammatory Response, and Gut Dysbiosis in Prenatal VPA‐Induced Rodent Model of Autism.” Physiology & Behavior 256: 113961. 10.1016/j.physbeh.2022.113961. 36100109

[jnc70316-bib-0002] Al‐Beltagi, M. 2021. “Autism Medical Comorbidities.” World Journal of Clinical Pediatrics 10: 15–28. 10.5409/wjcp.v10.i3.15. 33972922 PMC8085719

[jnc70316-bib-0003] Becker, K. G. 2012. “Male Gender Bias in Autism and Pediatric Autoimmunity: Male Bias in Autism and Pediatric Autoimmunity.” Autism Research 5: 77–83. 10.1002/aur.1227. 22431266 PMC4530611

[jnc70316-bib-0004] Bjørklund, G. , N. A. Meguid , M. A. El‐Bana , et al. 2020. “Oxidative Stress in Autism Spectrum Disorder.” Molecular Neurobiology 57: 2314–2332. 10.1007/s12035-019-01742-2. 32026227

[jnc70316-bib-0005] Boeing, T. , P. De Souza , S. Speca , et al. 2020. “Luteolin Prevents Irinotecan‐Induced Intestinal Mucositis in Mice Through Antioxidant and Anti‐Inflammatory Properties.” British Journal of Pharmacology 177: 2393–2408. 10.1111/bph.14987. 31976547 PMC7174882

[jnc70316-bib-0006] Bradley, P. P. , D. A. Priebat , R. D. Christensen , and G. Rothstein . 1982. “Measurement of Cutaneous Inflammation: Estimation of Neutrophil Content With an Enzyme Marker.” Journal of Investigative Dermatology 78: 206–209. 10.1111/1523-1747.ep12506462. 6276474

[jnc70316-bib-0007] Camuesco, D. , M. E. Rodríguez‐Cabezas , N. Garrido‐Mesa , et al. 2012. “The Intestinal Anti‐Inflammatory Effect of Dersalazine Sodium Is Related to a Down‐Regulation in IL‐17 Production in Experimental Models of Rodent Colitis.” British Journal of Pharmacology 165: 729–740. 10.1111/j.1476-5381.2011.01598.x. 21790535 PMC3315044

[jnc70316-bib-0008] Candelli, M. , L. Franza , G. Pignataro , et al. 2021. “Interaction Between Lipopolysaccharide and Gut Microbiota in Inflammatory Bowel Diseases.” International Journal of Molecular Sciences 22: 6242. 10.3390/ijms22126242. 34200555 PMC8226948

[jnc70316-bib-0009] Chaidez, V. , R. L. Hansen , and I. Hertz‐Picciotto . 2014. “Gastrointestinal Problems in Children With Autism, Developmental Delays, or Typical Development.” Journal of Autism and Developmental Disorders 44: 1117–11127. 10.1007/s10803-013-1973-x. 24193577 PMC3981895

[jnc70316-bib-0010] Chaliha, D. , M. Albrecht , M. Vaccarezza , et al. 2020. “A Systematic Review of the Valproic‐Acid‐Induced Rodent Model of Autism.” Developmental Neuroscience 42: 12–48. 10.1159/000509109. 32810856

[jnc70316-bib-0011] Chandler, S. , I. Carcani‐Rathwell , T. Charman , et al. 2013. “Parent‐Reported Gastro‐Intestinal Symptoms in Children With Autism Spectrum Disorders.” Journal of Autism and Developmental Disorders 43: 2737–2747. 10.1007/s10803-013-1768-0. 23371507

[jnc70316-bib-0012] Costa, R. A. , J. A. Amatnecks , G. O. Guaita , C. A. J. Stern , L. G. S. Branco , and A. R. Zampronio . 2024. “Sexual Dimorphism of Hypothalamic Serotonin Release During Systemic Inflammation: Role of Endothelin‐1.” Journal of Neuroimmunology 394: 578427. 10.1016/j.jneuroim.2024.578427. 39116522

[jnc70316-bib-0013] Da Silva, L. M. , A. Allemand , D. A. G. B. Mendes , et al. 2013. “Ethanolic Extract of Roots From *Arctium lappa* L. Accelerates the Healing of Acetic Acid‐Induced Gastric Ulcer in Rats: Involvement of the Antioxidant System.” Food and Chemical Toxicology 51: 179–187. 10.1016/j.fct.2012.09.026. 23036453

[jnc70316-bib-0014] Dargenio, V. N. , C. Dargenio , S. Castellaneta , et al. 2023. “Intestinal Barrier Dysfunction and Microbiota‐Gut‐Brain Axis: Possible Implications in the Pathogenesis and Treatment of Autism Spectrum Disorder.” Nutrients 15: 1620. 10.3390/nu15071620. 37049461 PMC10096948

[jnc70316-bib-0015] de Theije, C. G. , P. J. Koelink , G. A. Korte‐Bouws , et al. 2014. “Intestinal Inflammation in a Murine Model of Autism Spectrum Disorders.” Brain, Behavior, and Immunity 37: 240–247. 10.1016/j.bbi.2013.12.004. 24321212

[jnc70316-bib-0016] Deckmann, I. , J. Santos‐Terra , M. Fontes‐Dutra , et al. 2021. “Resveratrol Prevents Brain Edema, Blood‐Brain Barrier Permeability, and Altered Aquaporin Profile in Autism Animal Model.” International Journal of Developmental Neuroscience 81: 579–604. 10.1002/jdn.10137. 34196408

[jnc70316-bib-0017] Elnahas, E. M. , S. A. Abuelezz , M. I. Mohamad , et al. 2021. “Validation of Prenatal Versus Postnatal Valproic Acid Rat Models of Autism: A Behavioral and Neurobiological Study.” Progress in Neuro‐Psychopharmacology & Biological Psychiatry 108: 110185. 10.1016/j.pnpbp.2020.110185. 33238165

[jnc70316-bib-0018] Fiorentino, M. , A. Sapone , S. Senger , et al. 2016. “Blood‐Brain Barrier and Intestinal Epithelial Barrier Alterations in Autism Spectrum Disorders.” Molecular Autism 7: 49. 10.1186/s13229-016-0110-z. 27957319 PMC5129651

[jnc70316-bib-0019] Fiorica‐Howells, E. , R. Hen , J. Gingrich , Z. Li , and M. D. Gershon . 2002. “5‐HT(2A) Receptors: Location and Functional Analysis in Intestines of Wild‐Type and 5‐HT(2A) Knockout Mice.” American Journal of Physiology. Gastrointestinal and Liver Physiology 282: G877–G893. 10.1152/ajpgi.00435.2001. 11960784

[jnc70316-bib-0020] Franco, C. , F. Bonomini , E. Borsani , S. Castrezzati , L. Franceschetti , and R. Rezzani . 2021. “Involvement of Intestinal Goblet Cells and Changes in Sodium Glucose Transporters Expression: Possible Therapeutic Targets in Autistic BTBR T^+^Itpr3^tf^/J Mice.” International Journal of Environmental Research and Public Health 18: 11328. 10.3390/ijerph182111328. 34769857 PMC8583041

[jnc70316-bib-0021] Gąssowska‐Dobrowolska, M. , M. Cieślik , G. A. Czapski , et al. 2020. “Prenatal Exposure to Valproic Acid Affects Microglia and Synaptic Ultrastructure in a Brain‐Region‐Specific Manner in Young‐Adult Male Rats: Relevance to Autism Spectrum Disorders.” International Journal of Molecular Sciences 18: 3576. 10.3390/ijms21103576. PMC7279050 32443651

[jnc70316-bib-0022] Ghosh, S. S. , J. Bie , J. Wang , and S. Ghosh . 2014. “Oral Supplementation With Non‐Absorbable Antibiotics or Curcumin Attenuates Western Diet‐Induced Atherosclerosis and Glucose Intolerance in LDLR−/− Mice—Role of Intestinal Permeability and Macrophage Activation.” PLoS One 9: e108577. 10.1371/journal.pone.0108577. 25251395 PMC4177397

[jnc70316-bib-0023] Goldim, M. P. S. , A. Della Giustina , and F. Petronilho . 2019. “Using Evans Blue Dye to Determine Blood‐Brain Barrier Integrity in Rodents.” Current Protocols in Immunology 126: e83. 10.1002/cpim.83. 31483106

[jnc70316-bib-0024] Gross, E. R. , M. D. Gershon , K. G. Margolis , Z. V. Gertsberg , Z. Li , and R. A. Cowles . 2012. “Neuronal Serotonin Regulates Growth of the Intestinal Mucosa in Mice.” Gastroenterology 143: 408–417.e2. 10.1053/j.gastro.2012.05.007. 22609381 PMC3687781

[jnc70316-bib-0025] Gu, Y. , Y. Han , S. Ren , et al. 2022. “Correlation Among Gut Microbiota, Fecal Metabolites and Autism‐Like Behavior in an Adolescent Valproic Acid‐Induced Rat Autism Model.” Behavioural Brain Research 417: 113580. 10.1016/j.bbr.2021.113580. 34555431

[jnc70316-bib-0026] Gu, Y. Y. , Y. Han , J. J. Liang , et al. 2021. “Sex‐Specific Differences in the Gut Microbiota and Fecal Metabolites in an Adolescent Valproic Acid‐Induced Rat Autism Model.” Frontiers in Bioscience‐Landmark 26: 1585–1598. 10.52586/5051. 34994172

[jnc70316-bib-0027] Gustafsson, J. K. , and M. E. V. Johansson . 2022. “The Role of Goblet Cells and Mucus in Intestinal Homeostasis.” Nature Reviews. Gastroenterology & Hepatology 19: 785–803. 10.1038/s41575-022-00675-x. 36097076

[jnc70316-bib-0028] Habig, W. H. , M. J. Pabst , G. Fleischner , Z. Gatmaitan , I. M. Arias , and W. B. Jakoby . 1974. “The Identity of Glutathione S‐Transferase B With Ligandin, a Major Binding Protein of Liver.” Proceedings of the National Academy of Sciences of the United States of America 71: 3879–3882. 10.1073/pnas.71.10.3879. 4139704 PMC434288

[jnc70316-bib-0029] Haida, O. , T. Al.Sagheer , A. Balbous , et al. 2019. “Sex‐Dependent Behavioral Deficits and Neuropathology in a Maternal Immune Activation Model of Autism.” Translational Psychiatry 9: 124. 10.1038/s41398-019-0457-y. 30923308 PMC6438965

[jnc70316-bib-0030] Herath, M. , S. Hosie , J. C. Bornstein , A. E. Franks , and E. L. Hill‐Yardin . 2020. “The Role of the Gastrointestinal Mucus System in Intestinal Homeostasis: Implications for Neurological Disorders.” Frontiers in Cellular and Infection Microbiology 10: 248. 10.3389/fcimb.2020.00248. 32547962 PMC7270209

[jnc70316-bib-0031] Hirsch, M. M. , I. Deckmann , M. Fontes‐Dutra , et al. 2018. “Behavioral Alterations in Autism Model Induced by Valproic Acid and Translational Analysis of Circulating microRNA.” Food and Chemical Toxicology 115: 336–343. 10.1016/j.fct.2018.02.061. 29510222

[jnc70316-bib-0032] Horie, H. , O. Handa , Y. Naito , et al. 2022. “Subepithelial Serotonin Reduces Small Intestinal Epithelial Cell Tightness via Reduction of Occluding Expression.” Turkish Journal of Gastroenterology 33: 74–79. 10.5152/tjg.2022.20691. PMC9128500 35040791

[jnc70316-bib-0033] Jaber, M. 2023. “Genetic and Environmental Mouse Models of Autism Reproduce the Spectrum of the Disease.” Journal of Neural Transmission 130: 425–432. 10.1007/s00702-022-02555-9. 36318343

[jnc70316-bib-0034] James, D. M. , R. A. Kozol , Y. Kajiwara , et al. 2019. “Intestinal Dysmotility in a Zebrafish (*Danio rerio*) *shank3a;shank3b* Mutant Model of Autism.” Molecular Autism 10: 2–15. 10.1186/s13229-018-0250-4. 30733854 PMC6357389

[jnc70316-bib-0035] Jiang, Y. , C. Zhang , and T. Wang . 2021. “bFGF Ameliorates Intestinal Mucosal Permeability and Barrier Function Through Tight Junction Proteins in Burn Injury Rats.” Burns 47: 1129–1136. 10.1016/j.burns.2020.11.004. 33422356

[jnc70316-bib-0036] Kamionkowski, S. , F. Shibli , S. Ganocy , and R. Fass . 2022. “The Relationship Between Gastroesophageal Reflux Disease and Autism Spectrum Disorder in Adult Patients in the United States, Neurogastroenterol.” Motil 34: e14295. 10.1111/nmo.14295. 34859933

[jnc70316-bib-0037] Kim, J. W. , C. S. Choi , K. C. Kim , et al. 2013. “Gastrointestinal Tract Abnormalities Induced by Prenatal Valproic Acid Exposure in Rat Offspring.” Toxicology Research 29: 173–179. 10.5487/TR.2013.29.3.173. PMC3877996 24386517

[jnc70316-bib-0038] Kim, K. C. , P. Kim , H. S. Go , et al. 2011. “The Critical Period of Valproate Exposure to Induce Autistic Symptoms in Sprague‐Dawley Rats.” Toxicology Letters 201: 137–142. 10.1016/j.toxlet.2010.12.018. 21195144

[jnc70316-bib-0039] König, J. , J. Wells , P. D. Cani , et al. 2016. “Human Intestinal Barrier Function in Health and Disease.” Clinical and Translational Gastroenterology 7: e196. 10.1038/ctg.2016.54. 27763627 PMC5288588

[jnc70316-bib-0040] Kreider, C. M. , S. Mburu , S. Dizdarevic , G. Garvan , and J. H. Elder . 2021. “Exploration of Relationships Among Clinical Gastrointestinal Indicators and Social and Sensory Symptom Severity in Children With Autism Spectrum Disorder.” Pediatric Reports 13: 594–604. 10.3390/pediatric13040071. 34842807 PMC8628911

[jnc70316-bib-0041] Lee, Y. H. , S. H. Jeon , S. H. Kim , et al. 2012. “A New Synthetic Chalcone Derivative, 2‐Hydroxy‐3′,5,5′‐Trimethoxychalcone (DK‐139), Suppresses the Toll‐Like Receptor 4‐Mediated Inflammatory Response Through Inhibition of the Akt/NF‐kappaB Pathway in BV2 Microglial Cells.” Experimental & Molecular Medicine 44: 369–377. 10.3858/emm.2012.44.6.042. 22382990 PMC3389075

[jnc70316-bib-0042] Li, J. , H. Wang , W. Qing , et al. 2022. “Congenitally Underdeveloped Intestine Drives Autism‐Related Gut Microbiota and Behavior.” Brain, Behavior, and Immunity 105: 15–26. 10.1016/j.bbi.2022.06.006. 35714916

[jnc70316-bib-0043] Li, S. , N. Zhang , W. Li , H. L. Zhang , and X. X. Wang . 2024. “Gastrointestinal Problems in a Valproic Acid‐Induced Rat Model of Autism: From Maternal Intestinal Health to Offspring Intestinal Function.” World Journal of Psychiatry 14: 1095–1105. 10.5498/wjp.v14.i7.1095. 39050201 PMC11262932

[jnc70316-bib-0044] Liu, F. , K. Horton‐Sparks , V. Hull , R. W. Li , and V. Martínez‐Cerdeño . 2018. “The Valproic Acid Rat Model of Autism Presents With Gut Bacterial Dysbiosis Similar to That in Human Autism.” Molecular Autism 9: 61. 10.1186/s13229-018-0251-3. 30555669 PMC6288876

[jnc70316-bib-0045] Liu, S. , H. Xi , X. Xue , et al. 2024. “ *Clostridium butyricum* Regulates Intestinal Barrier Function via trek1 to Improve Behavioral Abnormalities in Mice With Autism Spectrum Disorder.” Cell & Bioscience 14: 95. 10.1186/s13578-024-01278-6. 39034406 PMC11265103

[jnc70316-bib-0046] Longo, B. , I. R. L. Andriolo , D. M. De Melo , M. M. De Souza , R. D. Prediger , and L. M. Da Silva . 2025. “Gastrointestinal Manifestations in Autism Spectrum and Attention‐Deficit/Hyperactivity Disorders: Pathogenesis and Drug Targets.” Current Developmental Disorders Reports 12: 1–14. 10.1007/s40474-025-00314-5.

[jnc70316-bib-0047] Lord, C. , T. S. Brugha , T. Charman , et al. 2020. “Autism Spectrum Disorder.” Nature Reviews Disease Primers 6, no. 1: 5. 10.1038/s41572-019-0138-4. PMC8900942 31949163

[jnc70316-bib-0048] Maisterrana, A. , F. De Chaumont , J. E. Longueville , E. Balado , E. Ey , and M. Jaber . 2024. “Female Mice Prenatally Exposed to Valproic Acid Exhibit Complex and Prolonged Social Behavior Deficits, Prog. Neuropsychopharmacol.” Biological Psychiatry 131: 110948. 10.1016/j.pnpbp.2024.110948. 38244714

[jnc70316-bib-0049] Mazefsky, C. , D. R. Schreiber , T. M. Olino , and N. J. Minshew . 2014. “The Association Between Emotional and Behavioral Problems and Gastrointestinal Symptoms Among Children With High‐Functioning Autism.” Autism 18: 493–501. 10.1177/1362361313485164. 24104507 PMC3980202

[jnc70316-bib-0050] McElhanon, B. O. , C. McCracken , S. Karpen , and W. G. Sharp . 2014. “Gastrointestinal Symptoms in Autism Spectrum Disorder: A Meta‐Analysis.” Pediatrics 133: 872–883. 10.1542/peds.2013-3995. 24777214

[jnc70316-bib-0051] Nicolini, C. , and M. Fahnestock . 2018. “The Valproic Acid‐Induced Rodent Model of Autism.” Experimental Neurology 299: 217–227. 10.1016/j.expneurol.2017.04.017. 28472621

[jnc70316-bib-0052] Percário, S. , A. VItal , and F. Jablonka . 1994. “Dosagem do malondialdeido.” Newslab 2: 46–50.

[jnc70316-bib-0053] Petersson, J. , O. Schreiber , G. C. Hansson , et al. 2011. “Importance and Regulation of the Colonic Mucus Barrier in a Mouse Model of Colitis.” American Journal of Physiology. Gastrointestinal and Liver Physiology 300: 327–333. 10.1152/ajpgi.00422.2010. PMC3302190 21109593

[jnc70316-bib-0054] Puricelli, C. , R. Rolla , L. Gigliotti , et al. 2022. “The Gut‐Brain‐Immune Axis in Autism Spectrum Disorders: A State‐Of‐Art Report.” Frontiers in Psychiatry 12: 755171. 10.3389/fpsyt.2021.755171. 35185631 PMC8850385

[jnc70316-bib-0055] Pusponegoro, H. D. , S. Ismael , S. Sastroasmoro , A. Firmansyah , and Y. Vandenplas . 2015. “Maladaptive Behavior and Gastrointestinal Disorders in Children With Autism Spectrum Disorder.” Pediatric Gastroenterology, Hepatology & Nutrition 18: 230–237. 10.5223/pghn.2015.18.4.230. PMC4712535 26770897

[jnc70316-bib-0056] Restrepo, B. , K. Angkustsiri , S. L. Taylor , et al. 2020. “Developmental‐Behavioral Profiles in Children With Autism Spectrum Disorder and Co‐Occurring Gastrointestinal Symptoms.” Autism Research 13: 1778–1789. 10.1002/aur.2354. 32767543 PMC7689713

[jnc70316-bib-0057] Salari, Z. , A. Moslemizadeh , S. S. Tezerji , et al. 2024. “Sex‐Dependent Alterations of Inflammatory Factors, Oxidative Stress, and Histopathology of the Brain‐Gut Axis in a VPA‐Induced Autistic‐Like Model of Rats.” Birth Defects Research 116: e2310. 10.1002/bdr2.2310. 38563145

[jnc70316-bib-0058] Schneider, T. , and R. Przewlocki . 2005. “Behavioral Alterations in Rats Prenatally Exposed to Valproic Acid: Animal Model of Autism.” Neuropsychopharmacology 30: 80–89. 10.1038/sj.npp.1300518. 15238991

[jnc70316-bib-0059] Sedlak, J. , and R. H. Lindsay . 1968. “Estimation of Total, Protein‐Bound, and Nonprotein Sulfhydryl Groups in Tissue With Ellman's Reagente.” Analytical Biochemistry 25: 192–205. 10.1016/0003-2697(68)90092-4. 4973948

[jnc70316-bib-0060] Sgritta, M. , S. W. Dooling , S. A. Buffington , et al. 2019. “Mechanisms Underlying Microbial‐Mediated Changes in Social Behavior in Mouse Models of Autism Spectrum Disorder.” Neuron 101: 246–259. 10.1016/j.neuron.2018.11.018. 30522820 PMC6645363

[jnc70316-bib-0061] Shimazu, R. , S. Akashi , H. Ogata , et al. 1999. “MD‐2, a Molecule That Confers Lipopolysaccharide Responsiveness on Toll‐Like Receptor 4.” Journal of Experimental Medicine 189: 1777–1782. 10.1084/jem.189.11.1777. 10359581 PMC2193086

[jnc70316-bib-0062] Tartaglione, A. M. , S. Schiavi , G. Calamandrei , and V. Trezza . 2019. “Prenatal Valproate in Rodents as a Tool to Understand the Neural Underpinnings of Social Dysfunctions in Autism Spectrum Disorder.” Neuropharmacology 159: 107477. 10.1016/j.neuropharm.2018.12.024. 30639388

[jnc70316-bib-0063] Thabault, M. , V. Turpin , A. Maisterrena , M. Jaber , M. Egloff , and L. Galvan . 2022. “Cerebellar and Striatal Implications in Autism Spectrum Disorders: From Clinical Observations to Animal Models.” International Journal of Molecular Sciences 23: 2294. 10.3390/ijms23042294. 35216408 PMC8874522

[jnc70316-bib-0064] Tutton, P. J. , and D. H. Barkla . 1980. “Neural Control of Colonic Cell Proliferation.” Cancer 45: 1172–1177. 10.1002/1097-0142(19800315)45:5+<1172::aid-cncr2820451322>3.0.co;2-b. 7357509

[jnc70316-bib-0065] Utrilla, M. P. , M. J. Peinado , R. Ruiz , et al. 2015. “Pea (*Pisum sativum* L.) Seed Albumin Extracts Show Anti‐Inflammatory Effect in the DSS Model of Mouse Colitis.” Molecular Nutrition & Food Research 59: 807–819. 10.1002/mnfr.201400630. 25626675

[jnc70316-bib-0066] Varley, A. N. , and K. N. Browning . 2024. “Gastrointestinal Dysfunction in the Valproic Acid Induced Model of Social Deficit in Rats.” Autonomic Neuroscience 253: 103161. 10.1016/j.autneu.2024.103161. 38461695 PMC11128350

[jnc70316-bib-0067] Vasconcelos, Q. D. J. S. , M. J. S. Frederico , R. S. Alves , T. J. P. G. Bandeira , M. E. A. Moraes , and G. F. Aragão . 2024. “Effects of Whey Protein Supplementation on Gut Microbiota of Wistar Rats With Valproic Acid‐Induced Autism Symptoms.” Future Microbiology 19: 213–226. 10.2217/fmb-2023-0051. 37934065

[jnc70316-bib-0068] Wu, X. X. , X. L. Huang , R. R. Chen , et al. 2019. “Paeoniflorin Prevents Intestinal Barrier Disruption and Inhibits Lipopolysaccharide (LPS)‐induced Inflammation in Caco‐2 Cell Monolayers.” Inflammation 42: 2215–2225. 10.1007/s10753-019-01085-z. 31473900

[jnc70316-bib-0069] Yang, X. , H. Li , C. Yang , and J. Ge . 2024. “Supplementation With Stigma Maydis Polysaccharide Attenuates Autism‐Like Behaviors and Improves Gut Function in Valproic Acid‐Induced Autism Model Male Rats.” International Journal of Developmental Neuroscience 84: 567–580. 10.1002/jdn.10354. 38923604

[jnc70316-bib-0070] Zhang, N. , S. T. Wang , and L. Yao . 2023. “Inhalation of *Cananga odorata* Essential Oil Relieves Anxiety Behaviors in Autism‐Like Rats via Regulation of Serotonin and Dopamine Metabolism.” Journal of Integrative Medicine 21: 205–214. 10.1016/j.joim.2023.01.006. 36792414

[jnc70316-bib-0071] Zhong, J. G. , W. T. Lan , Y. Q. Feng , et al. 2023. “Associations Between Dysbiosis Gut Microbiota and Changes of Neurotransmitters and Short‐Chain Fatty Acids in Valproic Acid Model Rats.” Frontiers in Physiology 14: 1077821. 10.3389/fphys.2023.1077821. 37035670 PMC10073564

