# Supplementary material for: Valproic Acid‐Induced Autistic‐Like Behavior Is Accompanied by Intestinal Damage Driving Changes in Gut Permeability in a Sex‐Dependent Way in Rats
Source: J Neurochem. 2025 Dec 15;169(12):e70316. doi: 10.1111/jnc.70316 (PMC12703684; doi:10.1111/jnc.70316)
Supplement: Supplementary file 1 — Appendix S1: jnc70316‐sup‐0001‐AppendixS1.zip. [file JNC-169-0-s001.zip › jnc70316-sup-0002-Supinfo1@Supplementary material.pdf]

Valproic acid-induced autistic-like behavior is accompanied by intestinal damage driving changes in gut permeability in a sex-dependently way in rats

Bruna Longo[a,b], Ruan Kaio Silva Nunes[b], Camila André Cazarin[b], Thiago Farias de Queiroz e Silva[b], Joanna Sievers[b], Ana Caroline dos Santos[b], Larissa Venzon[b], Levy Mota da Silva[b], Caio Henrique Willrich[a], Benhur Judah Cury[a], Regina Azevedo Costa[c], Márcia Maria de Souza[b], Cristina Aparecida Jark Stern[c], Aleksander Roberto Zampronio[c], Luisa Mota da Silva[a*].

[a]Department of Pharmacology, Federal University of Santa Catarina, Florianópolis, Santa Catarina, Brazil.

[b]Postgraduate in Pharmaceutical Sciences, University of Vale do Itajaí, Itajaí, Santa Catarina, Brazil.

[c]Department of Pharmacology, Federal University of Paraná, Curitiba, Paraná, Brazil.

*** Corresponding author.**

Tel.: +55 47 99931-9431

e-mail: lu.isamota@hotmail.com

Supplementary material

Prenatal exposure to VPA alters reproductive performance in female rats

Prenatal VPA exposure led to a 22% reduction in the number of pups per litter compared with saline-treated (11 ± 0.4 pups per litter) dams (normality test p=0.5, p=0.006, Fig. 1A). In the control group, litters consisted of an average of 4.7 ± 2.2 males and 5.8 ± 2.5 females. In contrast, litters from dams exposed to VPA *in utero* contained an average of 5.1 ± 1.6 males and 3.2 ± 1.2 females per litter (normality test p=0.3, $F_{(1, 16)}$=7.04, p=0.008, Fig. 1B). Furthermore, pup body weight at postnatal day (PND 7) was reduced by 16% and 22% in VPA-exposed males (15 ± 2 grams) and females (15 ± 1 grams), respectively, compared with the control group (normality test p=0.8, $F_{(1, 34)}$=3.39, p<0.0001, Fig. 1C).

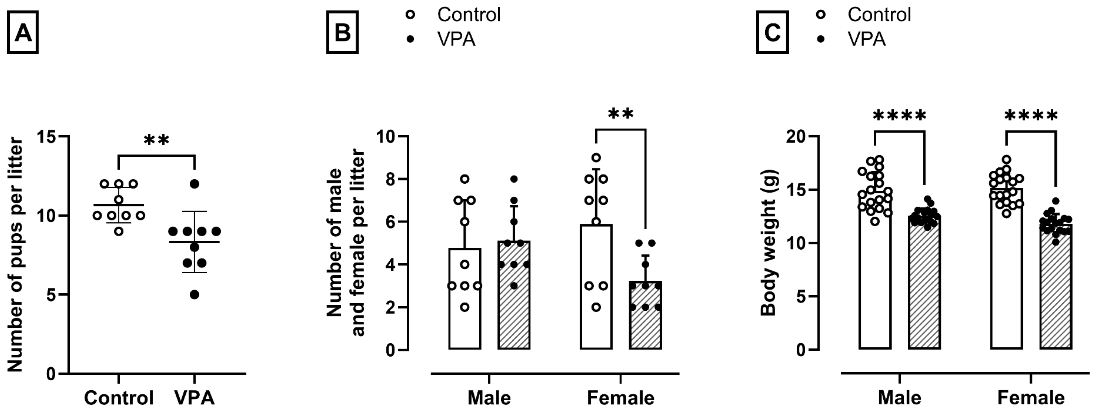

Supplemental Figure 1. Effects of prenatal exposure to VPA on the number of pups per litter (Panel A), number of males and females per litter (Panel B), and body weight of offspring on postnatal day (PND) 7 (Panel C). The results were expressed as means ± SD and the data were analyzed using the Student's *t*-test (Panel A, n = 9 rats per group) or two-way ANOVA followed by the Sidak's multiple comparison test (Panel B and C, n = 9 or 18, respectively). **p < 0.01 or ****p < 0.0001 when compared to the control group. VPA: valproic acid.
