# Supplementary figures and images for: Valproic Acid‐Induced Autistic‐Like Behavior Is Accompanied by Intestinal Damage Driving Changes in Gut Permeability in a Sex‐Dependent Way in Rats
Source: J Neurochem. 2025 Dec 15;169(12):e70316. doi: 10.1111/jnc.70316 (PMC12703684; doi:10.1111/jnc.70316)

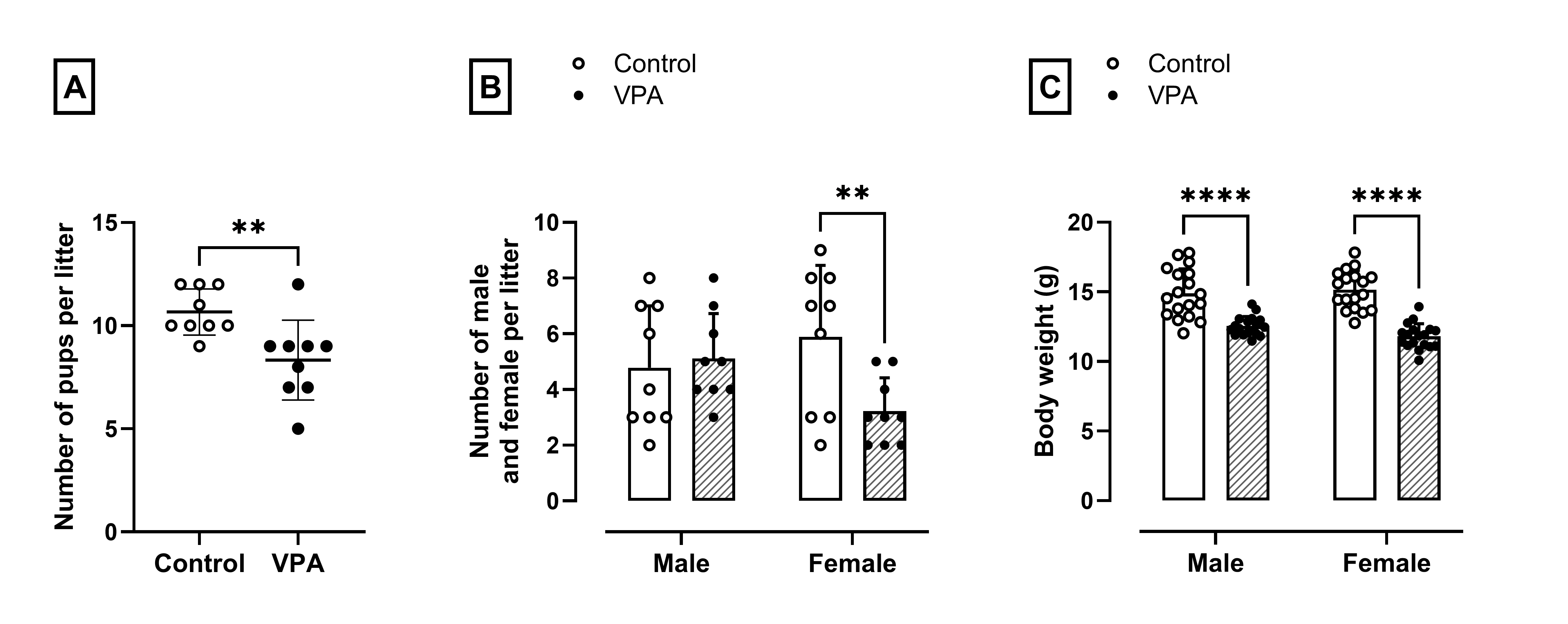

Supplement: Supplementary file 1 — Appendix S1: jnc70316‐sup‐0001‐AppendixS1.zip. [file JNC-169-0-s001.zip › jnc70316-sup-0001-FigureS1@Fig. 1 Supplementary Data.tif]
